# DR5 Governs Compound Exocytosis in Colonic Goblet Cells via TATA‐Box Binding Protein‐Dependent Bestrophin‐2 Transcriptional Regulation

**DOI:** 10.1002/advs.202516789

**Published:** 2025-11-11

**Authors:** Ying Wang, Xinyun Li, Yong Wang, Chuhe Yang, Xiaopei Gao, Ke Zhu, Yihang Ren, Jingxin Li, Chuanyong Liu, Bing Xue

**Affiliations:** ^1^ Department of Physiology and Pathophysiology School of Basic Medical Science Cheeloo College of Medicine Shandong University Jinan 250012 China; ^2^ State Key Laboratory for Innovation and Transformation of Luobing Theory Key Laboratory of Cardiovascular Remodeling and Function Research of MOE NHC CAMS and Shandong Province Department of Cardiology Qilu Hospital of Shandong University Jinan 250012 China

**Keywords:** Bestrophin‐2, compound exocytosis, death receptor 5, goblet cells, mucus barrier

## Abstract

The colonic mucus barrier, dependent on goblet cell‐secreted mucin2 (Muc2), prevents microbial invasion. Compound exocytosis enables the rapid, high‐volume release of mucus from goblet cells; its underlying mechanisms, however, remain unclear. Here, death receptor 5 (DR5) is identified as a critical regulator of this process. Knockout of *DR5* exhibits a thinner mucus layer despite elevated Muc2 and enlarged granules, causing mild dysbiosis and enhances susceptibility to *Citrobacter rodentium*. *DR5* knockout and its ligand *TRAIL* knockdown inhibits carbachol‐triggered goblet cell compound exocytosis, while DR5‐activator Bioymifi potentiates it. DR5 modulates intracellular pH through the colonic goblet cell‐expressed HCO_3_^−^ channel Bestrophin‐2 (Best2). TRAIL/DR5/Best2 co‐localize in goblet cells, and *Best2* knockdown abolishes DR5's pro‐exocytosis effect. Proteomics and bioinformatic analyses implicate TATA‐box binding protein (TBP) in the death domain‐dependent transcriptional regulation of *Best2* by DR5. This is supported by TBP‐*BEST2* promoter binding, as well as enhanced *BEST2* mRNA expression and promoter activity upon *TBP* overexpression. *DR5* knockout suppresses TBP expression in colonic goblet cells, while activation increases it. Moreover, *TBP* knockdown abrogates Bioymifi‐enhanced Best2 expression and compound exocytosis. The findings demonstrate that disruption of the DR5‐TBP‐Best2 axis in goblet cells perturbs goblet cell compound exocytosis and mucus layer formation, resulting in dysbiosis and heightened infection susceptibility.

## Graphical abstract

*DR5* knockout exhibits a thinner colonic mucus layer, leading to mild dysbiosis, increased susceptibility to *Citrobacter rodentium* invasion, and exacerbated epithelial injury. Mechanistically, DR5 binds transcription factor TBP, which regulates the expression of gene *Best2* via its promoter. This interaction influences goblet cell exocytosis and is essential for maintaining the integrity of the colonic mucus barrier.

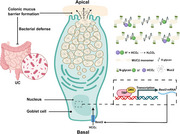

## 1 Introduction

Goblet cells, the predominant secretory epithelial cells in the colon, produce and secrete mucins to form the protective mucus layer overlying the intestinal epithelium.^[^ ^1^ ^]^ To withstand the heightened microbial burden, the colon maintains a unique two‐layered mucus structure: an outer loose layer hosting commensal microbes and an inner dense layer firmly adherent to the epithelium that prevents bacterial invasion and preserves sterility.^[^ ^2^ ^]^ Impairment of this barrier results in bacterial penetration into the inner mucus layer, a hallmark and potential trigger of ulcerative colitis (UC) and enteric infection.^[^ ^3^, ^4^, ^5^ ^]^

The gel‐forming, O‐linked glycosylated mucin2 (Muc2) constitutes the primary structural component of the mucus layer.^[^ ^6^ ^]^ Muc2 deficiency results in aberrant bacterial‐epithelial contact and spontaneous colitis.^[^ ^7^, ^8^ ^]^ Mucus barrier formation is a continuous process starting with Muc2 synthesis and packaging into secretory vesicles, followed by vesicle exocytosis and rapid hydration‐driven expansion.^[^ ^9^ ^]^ The efficiency of both exocytosis and the subsequent mucin expansion is critically dependent on bicarbonate (HCO_3_^−^) secretion and tightly regulated pH conditions.^[^ ^10^, ^11^, ^12^ ^]^ Two secretory modes govern mucus dynamics: basal secretion maintains continuous fusion and release of single mucin granules, while compound exocytosis is triggered by microbial or inflammatory stimuli where mucin vesicles rapidly fuse and empty their content.^[^ ^13^, ^14^, ^15^ ^]^ Therapeutic strategies targeting mucus dynamics are a promising frontier in UC management, while molecular regulators governing goblet cell secretion, specifically the ion channels that control the HCO_3_^−^ transport essential for these processes, remain incompletely defined.

Death receptor 5 (DR5), a member of the tumor necrosis factor receptor superfamily, comprises an extracellular domain, transmembrane region, and cytoplasmic death domain (DD).^[^ ^16^ ^]^ Besides pro‐apoptotic effect, DR5 activation by its ligand tumor necrosis factor‐related apoptosis‐inducing ligand (TRAIL) exhibits non‐apoptotic functions, such as pro‐inflammatory, pro‐survival, and proliferative roles.^[^ ^17^, ^18^ ^]^ In the human colon, TRAIL and DR5 localized to the upper parts of the crypts and the surface epithelium, key defensive sites against luminal pathogens.^[^ ^19^ ^]^ Our previous study has revealed the non‐apoptotic effect of DR5 in small intestinal epithelial cells (IECs): *DR5* knockout impaired the activity of intestinal stem cells and reduced goblet cell number in the ileum.^[^ ^20^ ^]^ Similarly, normal colonic epithelium was resistant to TRAIL‐induced apoptosis.^[^ ^21^ ^]^ However, DR5's physiological functions in the colon remain elusive. Prior work has shown that *DR5* knockout (*DR5* ^−/−^) mice are more susceptible to dextran sodium sulfate (DSS) induced colitis, an effect independent of DR5's pro‐apoptotic effect.^[^ ^22^ ^]^ Meanwhile, our preliminary data identified a significant thinning of the colonic mucus layer in *DR5* ^−/−^ mice, suggesting a potential defect in goblet cell function. Therefore, this study was designed to define the role of DR5 in colonic mucus layer formation and to uncover the key mechanisms responsible.

## 2 Results

### 2.1 *DR5* Knockout Disrupts Colonic Mucus Barrier Homeostasis

**Figure 1:**
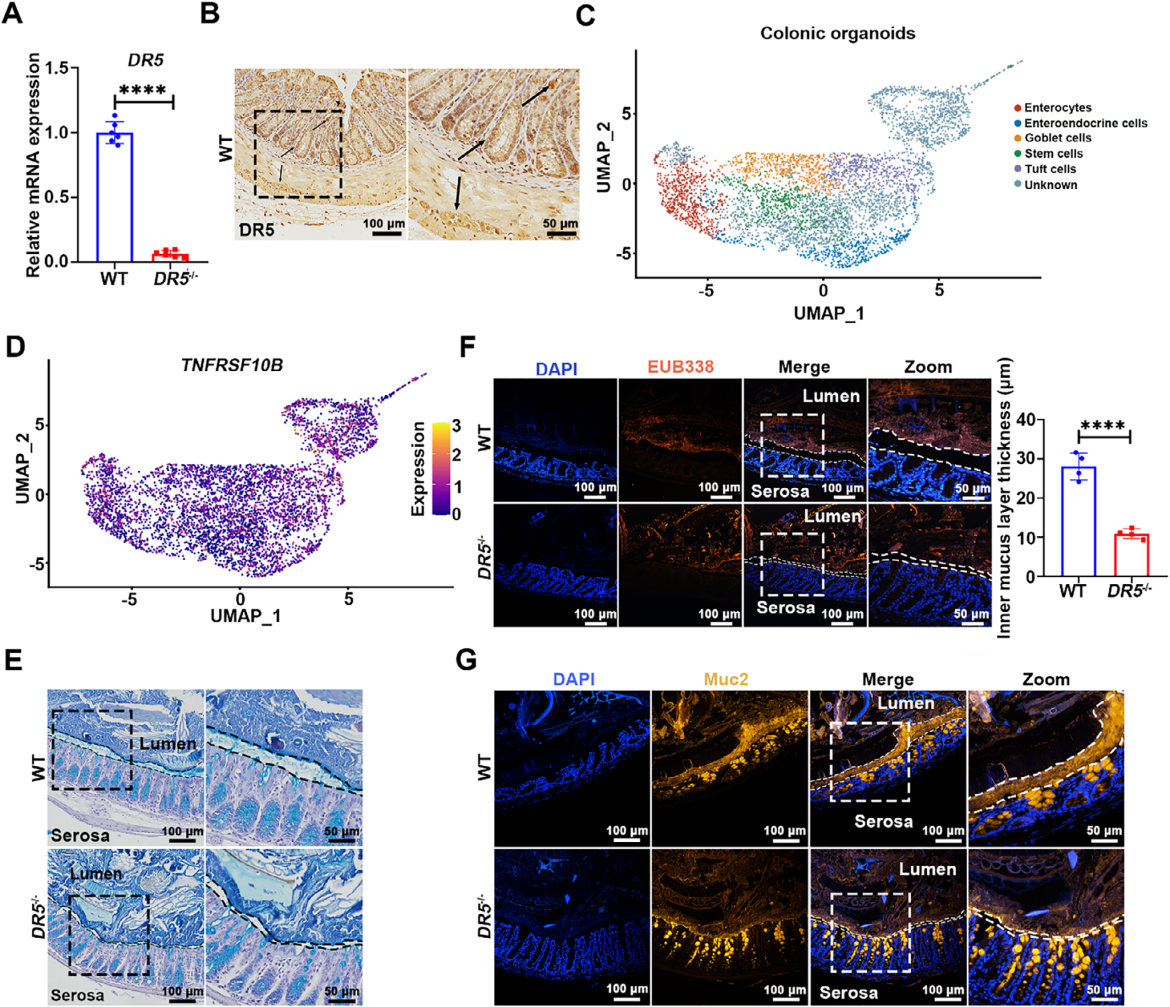
*DR5* knockout disrupts colonic mucus barrier homeostasis. A) Confirmation of *DR5* gene knockout in the colon of *DR5* ^−/−^ mice by quantitative real‐time PCR (qRT‐PCR) (*n* = 6). B) Images of immunohistochemical staining showing DR5 expression in the colon of WT mice. The black arrows indicate DR5‐positive cells. C) UMAP plot of single‐cell RNA sequencing data from human primary colonic organoids (GEO: GSE308421), depicting annotated cell types. D) UMAP plot illustrating expression levels of *TNFRSF10B* (encoding DR5) across the cellular landscape. E) AB‐stained Carnoy's‐fixed colon sections showing colonic inner mucus layer. The inner mucus layer (dashed lines) is thinner in *DR5* ^−/−^ mice compared with WT mice. F) Representative immunofluorescence images of Carnoy's‐fixed colon sections stained with DAPI (nuclei, blue) and the universal bacterial probe EUB338 (bacteria, red), with corresponding quantification of mucus layer thickness. White dashed lines outline the inner mucus layer. Quantification of inner mucus layer thickness from >10 randomly selected fields of view per mouse (200 × magnification; *n* = 4). G) Immunofluorescence images of Muc2 in Carnoy's‐fixed colon sections from WT and *DR5* ^−/−^ mice. White dashed lines outline the inner mucus layer. Data are expressed as mean ± SD. All data were analyzed by an unpaired *t*‐test. ^****^ *p* <0.0001.

First, we verified that the expression of *DR5* mRNA in the colon of *DR5* ^−/−^ mice was significantly lower than that of WT mice (**Figure** **1**A). Immunohistochemical staining localized DR5 protein predominantly to the colonic epithelium (Figure 1B). Consistent with this localization, analysis of a public single‐cell RNA‐sequencing dataset of human primary colonic organoids revealed ubiquitous *TNFRSF10B* (encoding DR5) expression across all five epithelial cell lineages, including goblet cells (Figure 1C,D). Histology showed pathological thinning of the inner mucus layer in *DR5* ^−/−^ mice, confirmed by Alcian blue (AB) staining and bacterial 16S rDNA fluorescence in situ hybridization (FISH) (Figure 1E,F). Consistent with this, *DR5* ^−/−^ mice had a significantly thinner Muc2 mucus layer in the colon (Figure 1G). These findings establish DR5 as an essential regulator of physiological Muc2‐dependent mucus barrier formation.

### 2.2 DR5 Governs Goblet Cell Compound Exocytosis through Ligand‐Dependent Signaling

**Figure 2:**
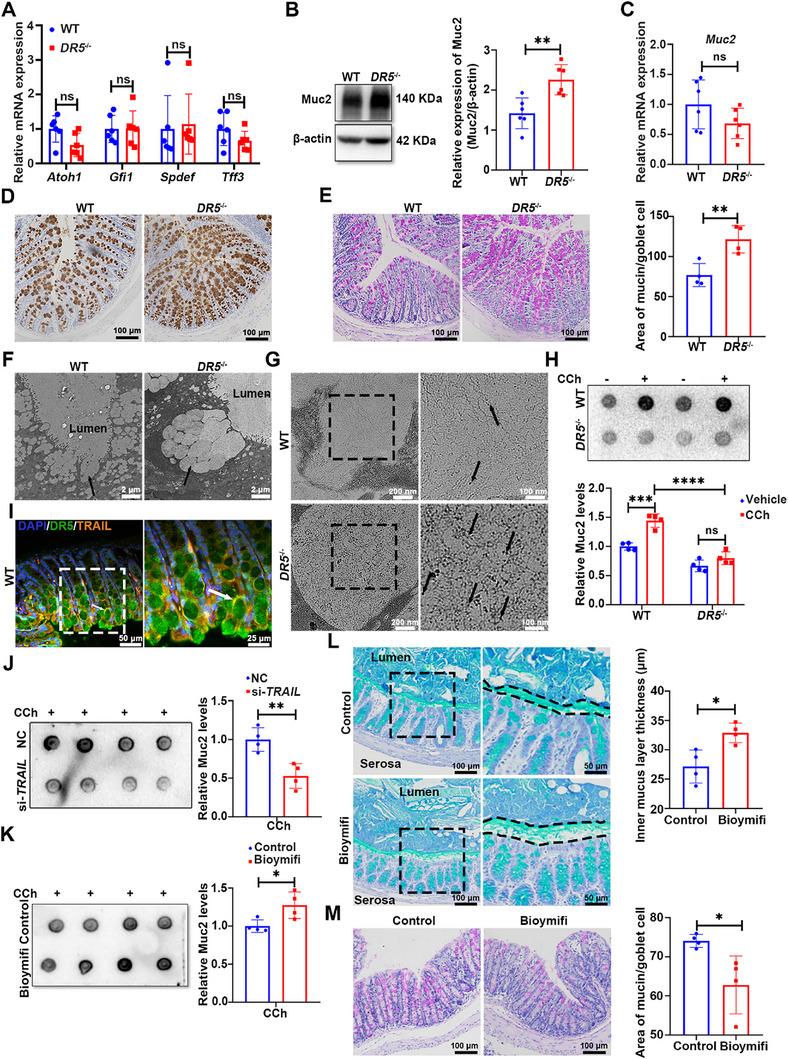
DR5 governs goblet cell compound exocytosis through ligand‐dependent signaling. A) qRT‐PCR analysis of goblet cell‐associated gene expression. Relative mRNA levels of key transcriptional regulators (*Atoh1*, *Gfi1*, *Spdef*) and the marker gene *Tff3* in the colons of WT and *DR5* ^−/−^ mice (*n* = 6). B) Western blot analysis of Muc2 protein levels in the colon of WT and *DR5* ^−/−^ mice (*n* = 6). C) *Muc2* mRNA expression in the colon of WT and *DR5* ^−/−^ mice quantified by qRT‐PCR (*n* = 6). D) Immunohistochemistry images of Muc2 in the colon of WT and *DR5* ^−/−^ mice. E) Representative images of periodic acid–Schiff (PAS)‐stained colon sections from WT and *DR5* ^−/−^ mice and corresponding statistical graph. Quantification of the average mucin area per goblet cell in >10 randomly selected crypts per mouse (*n* = 4). F,G) Transmission electron microscopy of colon sections. Black arrows indicate goblet cell vesicles (F) and granules within the vesicles (G). H) Representative images of Muc2 dot blotting in the supernatants of colonic organoids (Upper) and semi‐quantitative analysis of Muc2 levels (Lower). Colonic organoids generated from WT and *DR5* ^−/−^ mouse colonic crypts were cultured for 5 days, followed by stimulation with CCh (100 µm) for 30 min to trigger Muc2 secretion (*n* = 4). I) Immunofluorescence images of DR5 and TRAIL co‐staining in colon sections. The white arrow indicates a goblet cell. J) Representative images of Muc2 dot blotting in the supernatants of colonic organoids and semi‐quantitative analysis of Muc2 levels. Colonic organoids cultured for 5 days were transfected with si‐*TRAIL* or negative control (NC), followed by stimulation with CCh (100 µm) for 30 min to trigger Muc2 secretion (*n* = 4). K) Representative images of Muc2 dot blotting in the supernatants of colonic organoids and semi‐quantitative analysis of Muc2 level. Colonic organoids cultured for 3 days were treated with Bioymifi (100 nm) for 48 h, followed by stimulation with CCh (100 µm) for 30 min to trigger Muc2 secretion (*n* = 4). L) Representative AB‐stained colon sections (left) with corresponding quantification of mucus layer thickness (right) from mice following a 7‐day treatment regimen (*n* = 4). M) Representative images of PAS‐stained colon sections (left) with corresponding statistical graph of mucin area per goblet cell (right) following a 7‐day treatment regimen (*n* = 4). For both panels (L,M), mice received daily intracolonic infusions of the selective DR5 agonist Bioymifi (100 nm, 200 µL) or vehicle control for 7 days. Data are expressed as mean ± SD. Data in Figure 2H were analyzed by one‐way ANOVA followed by Tukey's post hoc test. Other data were analyzed by an unpaired *t*‐test. Ns indicates not significant. ^*^ *p* <0.05, ^**^ *p* <0.01, ^***^ *p* <0.001, ^****^ *p* <0.0001.

The thickness of the colonic mucus layer is dynamically regulated by Muc2 synthesis, post‐translational modification, and secretion.^[^ ^1^ ^]^ To determine whether the thinning of the mucus layer in *DR5* ^−/−^ mice stemmed from impaired goblet cell development, we analyzed the expression of key markers associated with goblet cell maturation and differentiation, including *Atoh1*, *Gfi1*, *Spdef*, and *Tff3*. No significant differences were observed between *DR5* ^−/−^ and WT colons, suggesting no defect in the differentiation and maturation of goblet cell (**Figure** **2**A). Notably, despite mucus layer thinning, Muc2 protein expression was significantly elevated in colonic goblet cells of *DR5* ^−/−^ mice compared to WT mice, whereas *Muc2* mRNA levels remained comparable between the two groups (Figure 2B,C). The intracellular Muc2 accumulation was further supported by an expanded Muc2‐positive area detected on immunohistochemistry (Figure 2D), enlarged cytoplasmic mucin domains (Figure 2E) in the colon of *DR5* ^−/−^ mice. Similarly, transmission electron microscopy showed that mucin granules in goblet cells of *DR5* ^−/−^ mice were significantly larger than those in WT mice (Figure 2F,G). Consistent with mucin over‐accumulation, high iron diamine/Alcian blue (HID/AB) staining indicated an increased area of both sialomucins and sulfomucins within goblet cells of *DR5* ^−/−^ mice, though the balance between these subtypes remained unchanged, indicating no major alterations in mucin quality (Figure S1, Supporting Information). Taken together, the paradoxical coexistence of Muc2 hyper‐accumulation within goblet cells and a thinned mucus layer, despite intact synthesis and maturation, suggests that the mucus defect in *DR5* ^−/−^ mice may arise from impaired mucin secretion.

Quantifying Muc2 content in supernatants of organoids enables evaluation of the secretory capacity of goblet cells and eliminates non‐epithelial confounders. Carbachol (CCh), a commonly used inducer of compound exocytosis in goblet cells, triggers rapid, high‐volume mucus release.^[^ ^23^ ^]^ Consistently, CCh treatment markedly increased Muc2 levels in organoid supernatants, confirming the induction of compound exocytosis. In contrast, this CCh‐induced Muc2 secretion was completely abolished in *DR5* ^−/−^ organoids (Figure S2A, Supporting Information; Figure 2H). DR5 and its ligand TRAIL co‐localized in colonic goblet cells (Figure 2I). Functionally, *TRAIL* knockdown in organoids significantly suppressed CCh‐induced Muc2 secretion in goblet cells, while TRAIL analogue Bioymifi (DR5 activator)^[^ ^24^ ^]^ potentiated it (Figure S2B, Supporting Information; Figure 2J,K). Moreover, in vivo administration of Bioymifi increased colonic mucus thickness and reduced the intracellular mucin area in goblet cells, indicating promoted mucin exocytosis (Figure 2L,M). No change was observed in Cleaved caspase‐3 expression in the colon of WT and *DR5* ^−/−^ mice, nor did the terminal deoxynucleotidyl transferase‐mediated dUTP nick‐end labeling (TUNEL) assay detect any excessive apoptosis in colonic goblet cells of *DR5* ^−/−^ mice (Figure S3, Supporting Information), ruling out an apoptotic mechanism under these conditions. Collectively, these results demonstrate that DR5 in IECs regulates goblet cell compound exocytosis via ligand‐dependent signaling to sustain the colonic mucus barrier.

### 2.3 DR5 Regulates Goblet Cell Compound Exocytosis via Bestrophin2‐Dependent Secretory Machinery

**Figure 3:**
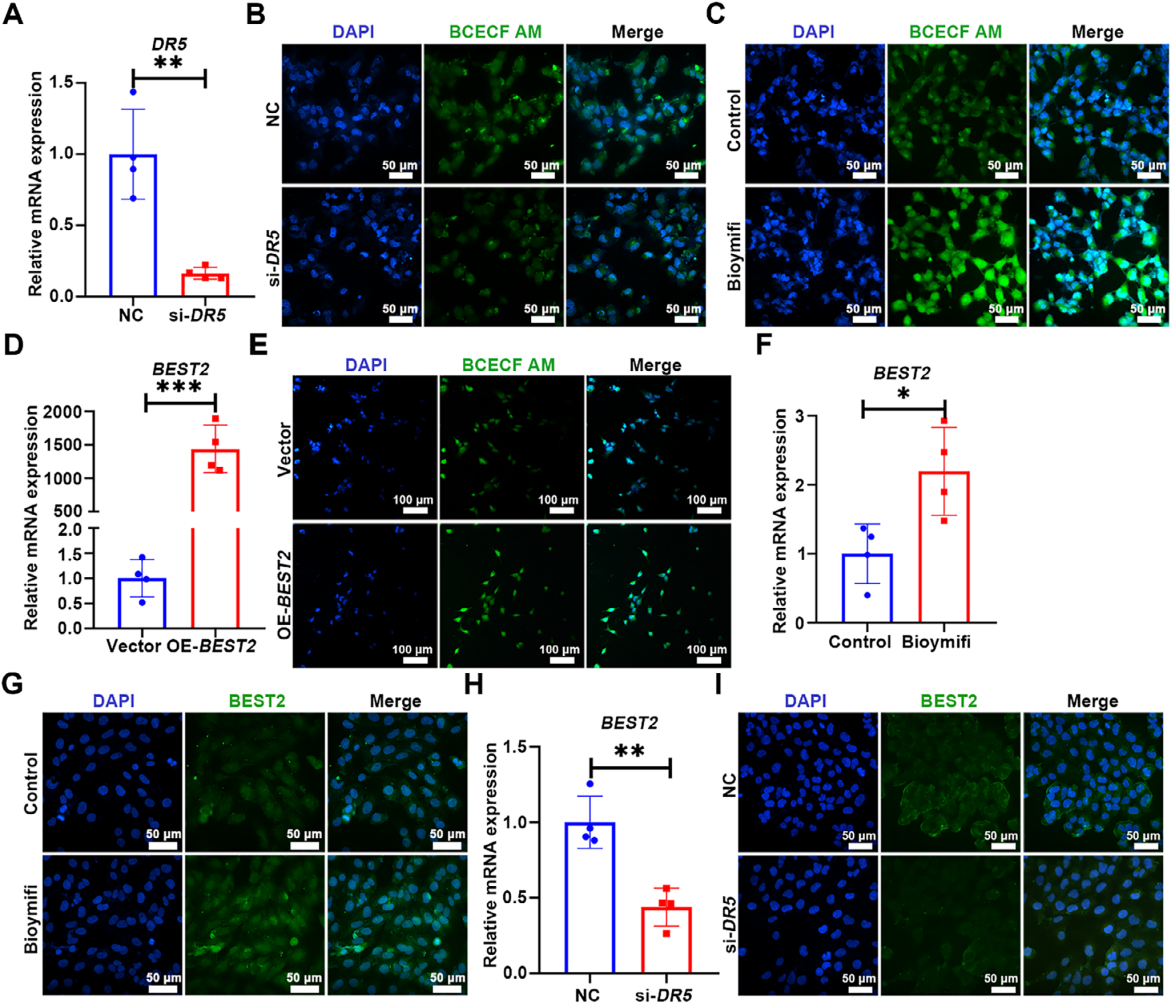
DR5 modulates intracellular pH via regulating BEST2 expression A) qRT‐PCR analysis of *DR5* knockdown efficiency in CCD 841 CoN cells transfected with si‐*DR5* or NC (*n* = 4). B) The effect of *DR5* knockdown on intracellular pH in CCD 841 CoN cells. Cells were transfected with si‐*DR5* or NC and then incubated with BCECF AM (1 µm) probe for 30 min. The lower the fluorescence intensity, the stronger the acidity. C) The effect of DR5 activation on intracellular pH in CCD 841 CoN cells. Cells were stimulated with Bioymifi (100 nm) for 48 h and then incubated with BCECF AM (1 µm) probe for 30 min. D) qRT‐PCR analysis of *BEST2* mRNA expression in CCD 841 CoN cells transfected with *BEST2* overexpression plasmid or empty vector (*n* = 4). E) The effect of *BEST2* overexpression on intracellular pH. CCD 841 CoN cells were transfected with *BEST2* overexpression plasmid or vector and then incubated with BCECF AM probe (1 µm) for 30 min. F) qRT‐PCR analysis of *BEST2* mRNA expression in CCD 841 CoN cells treated with Bioymifi (100 nm, 48 h) (*n* = 4). G) Immunofluorescence images of BEST2 protein in CCD 841 CoN cells stimulated with Bioymifi (100 nm, 48 h). H) qRT‐PCR analysis of *BEST2* mRNA expression in CCD 841 CoN cells transfected with si‐*DR5* or NC (*n* = 4). I) Immunofluorescence images of BEST2 protein in CCD 841 CoN cells transfected with si‐*DR5* or NC. Data are expressed as mean ± SD. All data were analyzed by an unpaired *t*‐test. ^*^ *p* <0.05, ^**^ *p* <0.01, ^***^ *p* <0.001.

Mucin exocytosis requires intracellular alkalinization to induce Muc2 depolymerization.^[^ ^9^ ^]^ Consistent with its role in Muc2 secretion, *DR5* knockdown induced intracellular acidification in CCD 841 CoN cells, a condition known to suppress secretory granule release.^[^ ^25^ ^]^ In contrast, Bioymifi induced alkalinization (**Figure** **3**A–C). Unlike in the colon, Muc2 levels in the small intestine of *DR5* ^−/−^ mice was significantly reduced,^[^ ^20^ ^]^ and there was no excessive accumulation of cytoplasmic mucin in goblet cells (Figure S4, Supporting Information). The colonic‐specific function of DR5 prompted our attention to Bestrophin‐2 (Best2), a channel specifically expressed in colonic goblet cells, mediates vectorial HCO_3_^−^ secretion.^[^ ^26^, ^27^ ^]^ In line with its established role, *BEST2* overexpression resulted in intracellular alkalinization in CCD 841 CoN cells (Figure 3D,E). DR5 dynamically controlled BEST2 expression in CCD 841 CoN cells: Bioymifi treatment upregulated *BEST2* mRNA and BEST2 protein expression, while *DR5* knockdown suppressed them (Figure 3F–I). These results indicated that BEST2 was the downstream effector molecule of DR5 in regulating intracellular pH.

**Figure 4:**
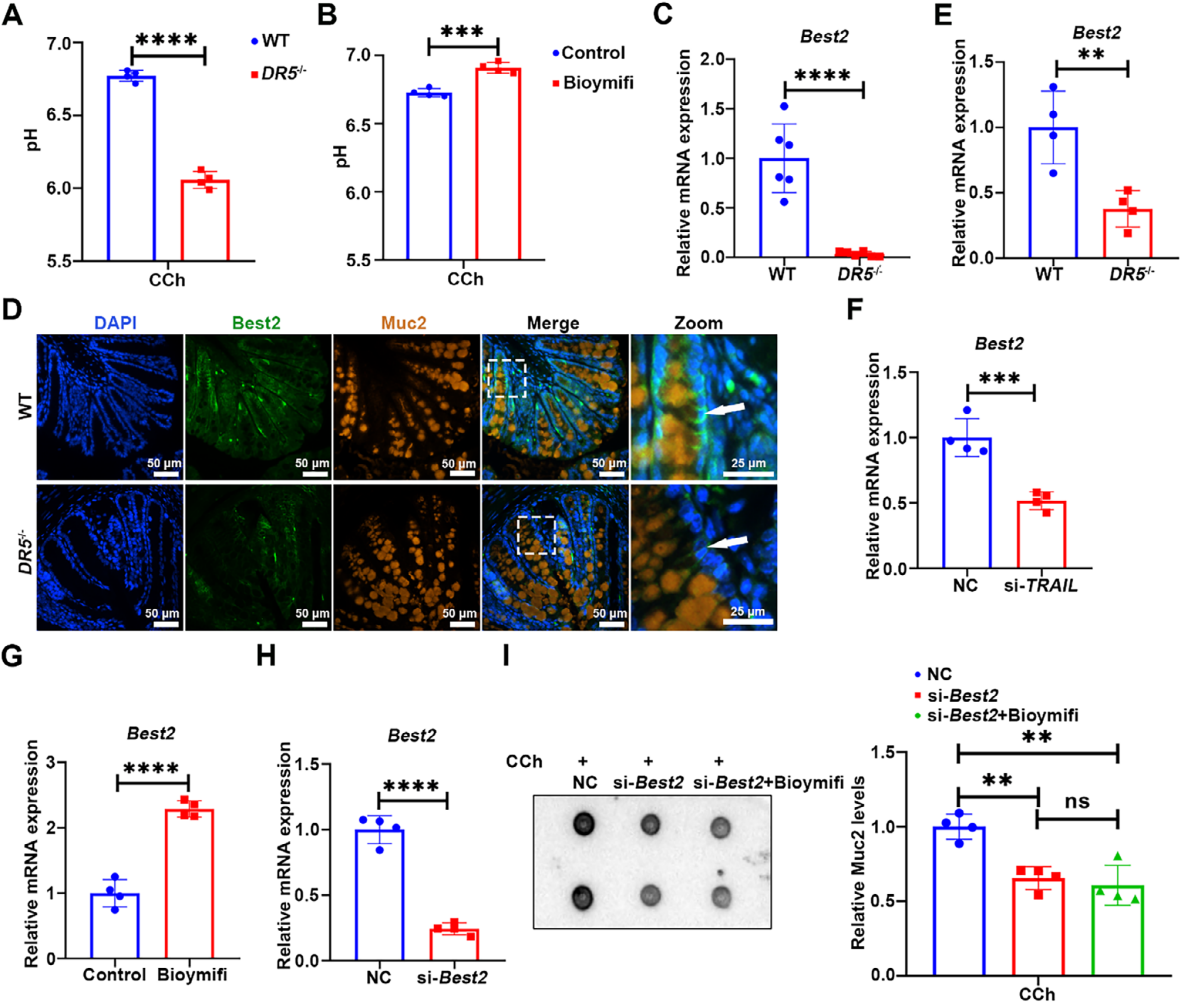
DR5 governs goblet cell compound exocytosis through a Best2‐dependent pathway. A) Supernatant pH in colonic explants from WT and *DR5* ^−/−^ mice (*n* = 4). pH was measured in the supernatant after a 10 min stimulation with CCh (1 mm). B) Supernatant pH in colonic explants from Bioymifi‐treated mice (*n* = 4). Mice were pretreated with Bioymifi (100 nm, 200 µL) or vehicle via colonic perfusion for 7 days. Supernatant pH was measured following a 10 min ex vivo challenge with CCh (1 mm). C) qRT‐PCR analysis of *Best2* mRNA expression in the colon of WT and *DR5* ^−/−^ mice (*n* = 6). D) Immunofluorescence images of Best2 in the colon of WT and *DR5* ^−/−^ mice. Goblet cells are marked by Muc2. The white arrow indicates Best2^+^ goblet cells. E) qRT‐PCR analysis of the *Best2* mRNA expression in colonic organoids from WT and *DR5* ^−/−^ mice (*n* = 4). F) Effect of *TRAIL* knockdown on the *Best2* mRNA expression in colonic organoids. Colonic organoids cultured for 5 days were transfected with si‐*TRAIL* or NC (*n* = 4). G) Effect of DR5 activation on the *Best2* mRNA expression in colonic organoids. Colonic organoids cultured for 3 days were treated with Bioymifi (100 nm) for 48 h to activate DR5 specifically in intestinal epithelium (*n* = 4). H) Confirmation of *Best2* knockdown efficiency in colonic organoids by qRT‐PCR. Colonic organoids cultured for 5 days were transfected with si‐*Best2* or NC (*n* = 4). I) Effect of Best2 on DR5‐mediated potentiation of Muc2 secretion in goblet cells. Representative images of Muc2 dot blotting in the supernatants of colonic organoids and semi‐quantitative analysis of Muc2 level. Colonic organoids cultured for 5 days were transfected with si‐*Best2* or NC, and then treated with or without Bioymifi (100 nm) for 48 h. Muc2 secretion was then induced by CCh (100 µm) for 30 min (*n* = 4). Data are expressed as mean ± SD. Data in Figure 4I was analyzed by one‐way ANOVA followed by Tukey's post hoc test. Other data were analyzed by an unpaired *t*‐test. Ns indicates not significant. ^**^ *p* <0.01, ^***^ *p* <0.001, ^****^ *p* <0.0001.

Best2 mediates the CCh‐activated, HCO_3_^−^‐dependent transepithelial current by establishing a directional HCO_3_^−^ flow, facilitating its basolateral uptake and apical efflux into the intestinal lumen, which is critical for pH homeostasis and mucin expansion.^[^ ^10^, ^26^, ^28^ ^]^ In colonic explants submerged in ultrapure water, CCh treatment triggered a rapid increase in supernatant pH (Figure S5, Supporting Information), indicative of luminal HCO_3_^−^ secretion that facilitates mucin secretion and expansion.^[^ ^11^ ^]^ However, this CCh‐induced response was significantly attenuated in colonic explants from *DR5* ^−/−^ mice compared to WT mice (**Figure** **4**A). Conversely, colonic perfusion with the DR5 agonist Bioymifi resulted in a significantly higher supernatant pH in response to CCh, compared to the control group (Figure 4B). *Best2* mRNA levels were reduced significantly in the colon of *DR5* ^−/−^ mice relative to WT mice, and its protein was similarly reduced in colonic goblet cells (Figure 4C,D). This phenotype was recapitulated in both *DR5* ^−/−^ organoids and *TRAIL* knockdown organoids (Figure 4E,F). Conversely, Bioymifi upregulated *Best2* mRNA expression in colonic organoids (Figure 4G). Critically, *Best2* knockdown not only inhibited CCh‐induced Muc2 secretion in goblet cells but also blocked Bioymifi's potentiating effects on Muc2 secretion (Figure 4H,I). Together, this coherent genetic and pharmacological evidence identifies Best2 as an essential downstream effector through which DR5 regulates compound exocytosis—a function likely mediated by the role of Best2 in intracellular pH regulation.

### 2.4 DD Activity is Necessary for DR5 Driving *BEST2* Transcription

**Figure 5:**
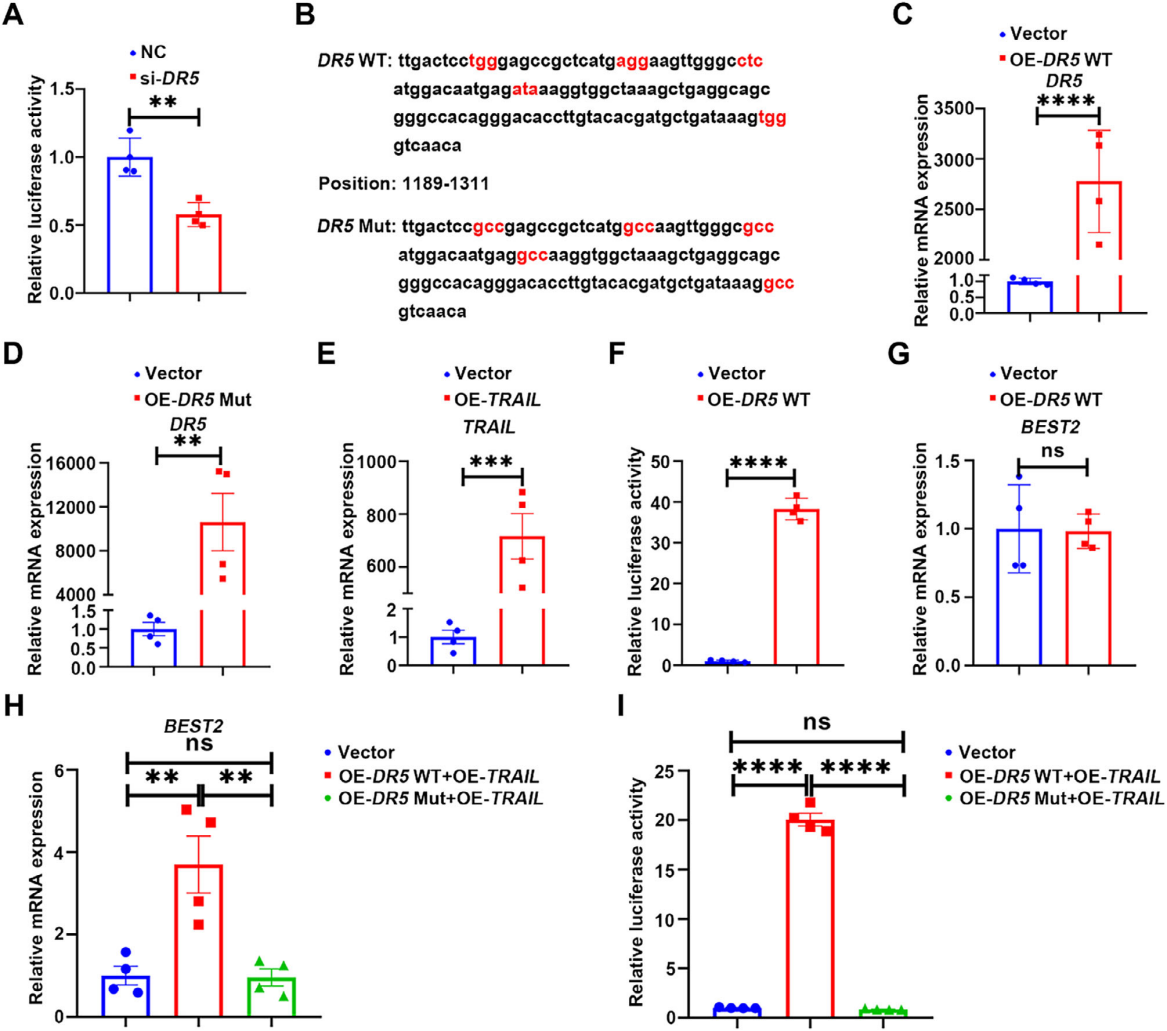
DR5 driving *BEST2* transcription requires its DD activity. A) Dual‐luciferase reporter assay assessing the effect of *DR5* knockdown on the *BEST2* promoter activity in CCD 841 CoN cells (*n* = 4). B) Mutation sites of the DR5 DD. Red refers to the mutated amino acid. C) qRT‐PCR analysis of *DR5* mRNA expression in HEK293T cells transfected with *DR5* WT plasmid or vector (*n* = 4). D) qRT‐PCR analysis of *DR5* mRNA expression in HEK293T cells transfected with *DR5* Mut plasmid or vector (*n* = 4). E) qRT‐PCR analysis of *TRAIL* mRNA expression in HEK293T cells transfected with *TRAIL* plasmid or vector (*n* = 4). F) Dual‐luciferase reporter assay showing the effect of *DR5* WT plasmid on *BEST2* promoter activity in HEK293T cells (*n* = 4). G) qRT‐PCR analysis of *BEST2* mRNA expression in HEK293T cells transfected with *DR5* WT plasmid or vector (*n* = 4). H) qRT‐PCR analysis of *BEST2* mRNA expression in HEK293T cells co‐transfected with *DR5* WT and *TRAIL* plasmids or *DR5* Mut and *TRAIL* plasmids (*n* = 4). I) Dual‐luciferase reporter assay showing the effect of co‐transfection with *DR5* WT and *TRAIL* plasmids or *DR5* Mut and *TRAIL* plasmids on *BEST2* promoter activity in HEK293T cells (*n* = 4). Data are expressed as mean ± SD. Data in Figure 5H,I were analyzed by one‐way ANOVA followed by Tukey's post hoc test. Other data were analyzed by an unpaired *t*‐test. Ns indicates not significant. ^**^ *p* <0.01, ^***^ *p* <0.001, ^****^ *p* <0.0001.

*DR5* knockdown in CCD 841 CoN cells significantly reduced *BEST2* promoter activity, suggesting transcriptional regulation by DR5 (**Figure** **5**A). Given that HEK293T cells exhibited lower expression of *DR5* and *TRAIL* compared to CCD 841 CoN cells, while also maintaining stable basal BEST2 expression (Figure S6A–C, Supporting Information), we established *DR5*/*TRAIL* overexpression models in HEK293T cells. Based on the established role of the DD in intracellular signaling of DR5 activation,^[^ ^29^ ^]^ HEK293T cells were transfected with plasmids encoding wild‐type *DR5* (*DR5* WT) or *DR5* carrying DD inactivating mutation (*DR5* Mut) and *TRAIL* (Figure 5B–E). Overexpression of *DR5* WT alone enhanced *BEST2* promoter activity without altering *BEST2* mRNA levels (Figure 5F,G). In contrast, co‐transfection of *DR5* WT with *TRAIL* significantly upregulated *BEST2* mRNA levels and its promoter activity (Figure 5H,I). These findings suggested that ligand‐bound DR5 induced sufficient signaling to amplify *BEST2* transcription, consistent with DR5's ligand‐dependent effect on promoting goblet cell Muc2 secretion observed above. Crucially, co‐transfection of *DR5* Mut and *TRAIL* failed to alter either *BEST2* mRNA levels or promoter activity, demonstrating functional DD activity was essential for TRAIL/DR5‐mediated *BEST2* transcriptional regulation (Figure 5H,I). Furthermore, chromatin immunoprecipitation (ChIP)‐qPCR assays detected no direct binding of DR5 to the *BEST2* promoter (Figure S6D, Supporting Information), indicating that DR5 regulated *BEST2* transcription indirectly, a process requiring functional DD.

### 2.5 DR5 Regulates *BEST2* Transcription via Modulation of Transcription Factor TATA‐Box Binding Protein Expression

**Figure 6:**
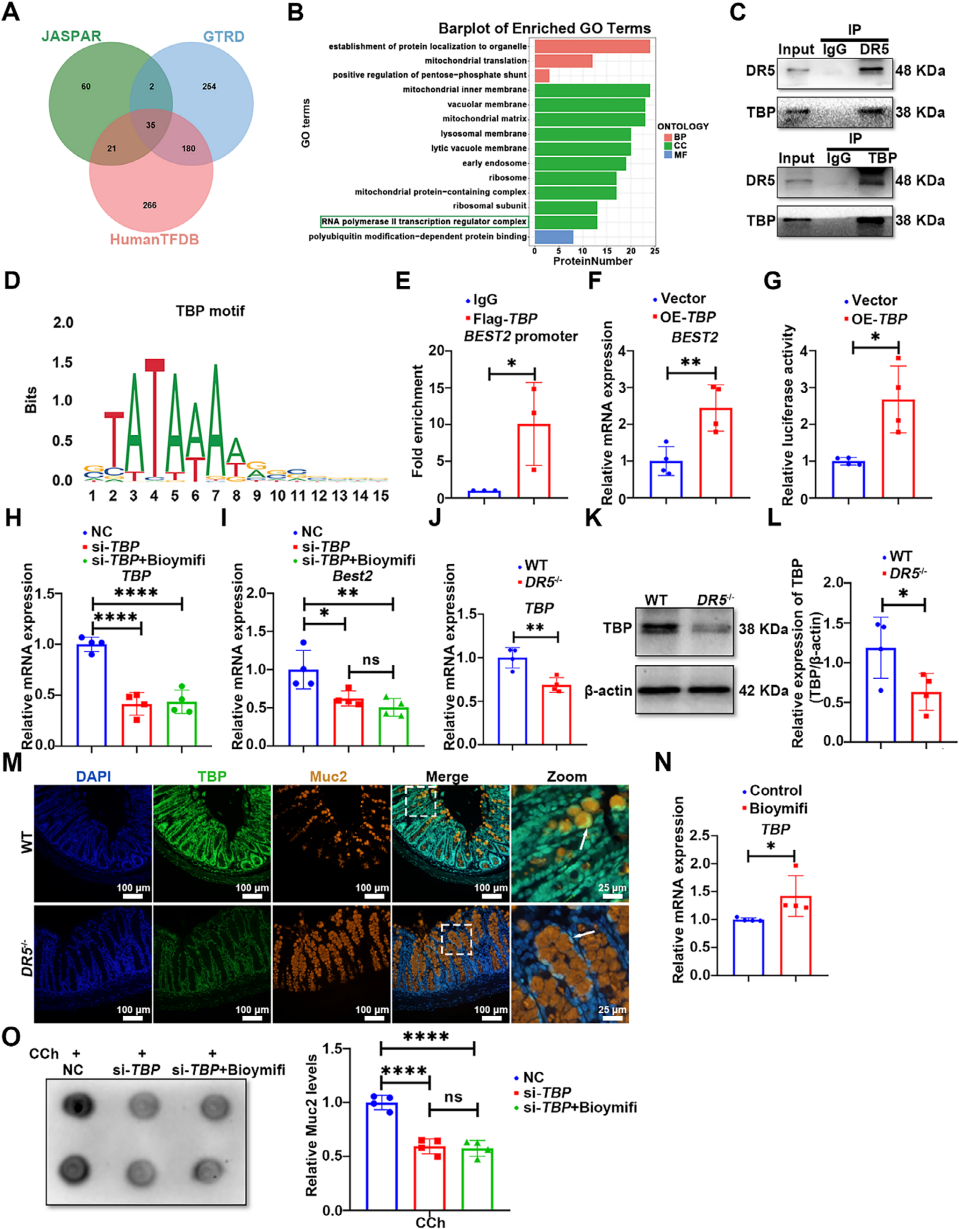
DR5 regulates *BEST2* transcription via modulation of transcription factor TBP expression. A) Bioinformatic analysis combined with Venn diagram analysis to screen common predicted transcription factors regulating *BEST2* transcription. B) Gene Ontology enrichment analysis of DR5‐interacting proteins (from co‐IP/MS) highlighting "RNA polymerase II transcription regulator complex" as a significantly enriched term. C) Reciprocal co‐IP assays in colonic crypt cells confirm the DR5‐TBP interaction. D) Predicted TBP‐binding motif within the *BEST2* promoter region (predicted using JASPAR database). E) ChIP‐qPCR analysis of TBP binding to the *BEST2* promoter in HEK293T cells transfected with Flag‐*TBP* plasmid. IP: anti‐Flag antibody (*n* = 3). F) qRT‐PCR analysis of *BEST2* mRNA expression in HEK293T cells transfected with *TBP* plasmid or vector (*n* = 4). G) Dual‐luciferase reporter assay showing the effect of *TBP* plasmid on *BEST2* promoter activity in HEK29T cells (*n* = 4). H) Confirmation of *TBP* knockdown efficiency in colonic organoids by qRT‐PCR. Colonic organoids cultured for 5 days were transfected with si‐*TBP* or NC, and then treated with or without Bioymifi (100 nm) for 48 h (*n* = 4). I) qRT‐PCR analysis of *Best2* mRNA expression in colonic organoids. Colonic organoids cultured for 5 days were transfected with si‐*TBP* or NC, and then treated with or without Bioymifi (100 nm) for 48 h (*n* = 4). J) qRT‐PCR analysis of *TBP* mRNA expression in colonic organoids of WT and *DR5* ^−/−^ mice (*n* = 4). K) and L) Protein level of TBP analyzed by western blot in colonic organoids of WT and *DR5* ^−/−^ mice (*n* = 4). M) Immunofluorescence images of TBP expression in colon sections of WT and *DR5* ^−/−^ mice. The white arrow indicates Muc2^+^ goblet cells. N) qRT‐PCR analysis of *TBP* mRNA expression in colonic organoids stimulated with Bioymifi (100 nm, 48 h) (*n* = 4). O) The effect of *TBP* knockdown on DR5 activation‐induced Muc2 secretion in goblet cells. Representative images of Muc2 dot blotting in the supernatants of colonic organoids and semi‐quantitative analysis of Muc2 level. Colonic organoids cultured for 5 days were transfected with si‐*TBP* or NC, and then treated with or without Bioymifi (100 nm) for 48 h. After the treatment, CCh (100 µm) was administrated for 30 min to induce Muc2 secretion in goblet cells (*n* = 4). Data are expressed as mean ± SD. Data in Figure 6H,I,O were analyzed by one‐way ANOVA by Tukey's post hoc test. Other data were analyzed by an unpaired *t*‐test. Ns indicates not significant. ^*^ *p* <0.05, ^**^ *p* <0.01, ^****^ *p* <0.0001.

To clarify the mechanism underlying DR5's transcriptional regulation of *BEST2*, we employed a multi‐tiered approach. We first identified candidate transcription factors (TFs) for *BEST2* by intersecting predictions from three independent TF databases (JASPAR, GTRD, HumanTFDB), yielding 35 high‐confidence candidates (Table S1, Supporting Information; **Figure** **6**A). In parallel, DR5 immunoprecipitation (IP) coupled with mass spectrometry (MS) in CCD 841 CoN cells identified DR5‐interacting proteins. Gene Ontology enrichment analysis of the DR5 interactome revealed significant enrichment for “RNA polymerase II (Pol II) transcription regulator complex” (Figure 6B). Among the candidate TFs, TATA‐box binding protein (TBP) was noticed, which is the DNA‐binding core of the transcription factor II D (TFIID) complex and provides the critical anchor point for assembling the Pol II pre‐initiation complex.^[^ ^30^ ^]^ Although TBP itself was not detected by IP‐MS, likely due to sensitivity limitation, multiple critical TFIID components were detected, including TBP‐related factor 4 (TAF4), TAF7, and TAF9, which are crucial for TFIID complex assembly and function.^[^ ^31^, ^32^ ^]^ Reciprocal co‐IP in colonic organoids confirmed a direct physical interaction between DR5 and TBP (Figure 6C), which likely occurred in the nucleus, as supported by their nuclear co‐localization in goblet cells (Figure S7A, Supporting Information). We next investigated whether TBP directly regulates *BEST2* transcription. We predicted TBP binding sites in the *BEST2* promoter using the JASPAR database; direct binding of TBP to *BEST2* promoter regions was subsequently validated by ChIP‐qPCR (Figure S7B, Supporting Information; Figure 6D,E). Consistently, *TBP* overexpression in HEK293T cells upregulated both *BEST2* mRNA levels and promoter activity, establishing TBP as a direct transcriptional activator of *BEST2* (Figure 6F,G). Critically, *TBP* knockdown in organoids markedly reduced basal *Best2* mRNA expression and abolished Bioymifi‐induced *Best2* mRNA upregulation (Figure 6H,I). We then examined whether TBP itself was regulated by DR5. *DR5* knockout significantly suppressed *TBP* expression at both the mRNA and protein levels in organoids (Figure 6J–L). In parallel to these findings, a pronounced reduction of nuclear TBP was observed in the colonic goblet cells of *DR5* ^−/−^ mice (Figure 6M). Conversely, DR5 activation by Bioymifi stimulated *TBP* mRNA and protein expression in organoids and CCD 841 CoN cells (Figure S7C,D, Supporting Information; Figure 6N). Functionally, *TBP* knockdown not only inhibited CCh‐induced Muc2 secretion in goblet cells but also blocked the potentiating effects of Bioymifi on Muc2 secretion in goblet cells (Figure 6O). Collectively, these results establish TBP as a key downstream effector through which DR5 controls *Best2* transcription and thereby regulates goblet cell compound exocytosis.

### 2.6 *DR5* Knockout Results in a Mild Colonic Dysbiosis under Steady‐State Conditions

**Figure 7:**
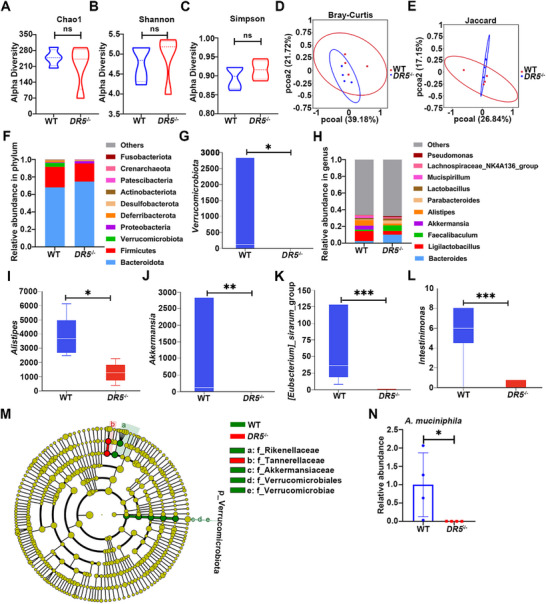
*DR5* knockout results in a mild colonic dysbiosis under steady‐state conditions. A–C) Alpha diversity (Chao1, Shannon, Simpson indices) analysis of gut microbiota in WT and *DR5* ^−/−^ mice (*n* = 4). D,E) Beta diversity analysis of gut microbiota based on Bray‐Curtis and Jaccard distances (*n* = 4). F) Phylum‐level taxonomic composition of gut microbiota in WT and *DR5* ^−/−^ mice (*n* = 4). G) Relative abundance of *Verrucomicrobia* in WT and *DR5* ^−/−^ mice (*n* = 4). H) Genus‐level taxonomic composition of gut microbiota in WT and *DR5* ^−/−^ mice (*n* = 4). I–L) Genus‐specific abundance: *Alistipes* (I), *Akkermansia* (J), *[Eubacterium]_siraeum*_group (K), and *Intestinimonas* (L) in WT and *DR5* ^−/−^ mice (*n* = 4). M) Differential bacterial biomarkers identified by LEfSe analysis from WT vs *DR5* ^−/−^ mice (Linear Discriminant Analysis [LDA] score >4, *p* <0.05) (*n* = 4). N) The abundance of *A. muciniphila* in fecal samples from WT and *DR5* ^−/−^ mice was analyzed by qPCR (*n* = 4). Data in Figure 7A,C were analyzed by an unpaired *t*‐test. Data in Figure 7B,N were analyzed by Mann–Whitney U tests. Differentially abundant taxa (Figure 7G,I–L) were identified using the ZIG model in the metagenomeSeq R package with *p‐*value <0.05. Ns indicates not significant. ^*^ *p* <0.05, ^**^ *p* <0.01, ^***^ *p* <0.001.

Given the critical role of an intact mucus layer in microbiota‐mucus interactions, we performed 16S rRNA sequencing to analyze the fecal microbiota. Alpha diversity (Chao1, Shannon, Simpson indices) and beta diversity (Bray‐Curtis and Jaccard distances) showed no significant difference between WT and *DR5* ^−/−^ mice (**Figure** **7**A–E). However, microbial composition differed: *DR5* ^−/−^ mice exhibited reduced *Verrucomicrobia* abundance, a phylum associated with mucosal homeostasis (Figure 7F,G).^[^ ^33^ ^]^ At the genus level, *DR5* ^−/−^ mice showed decreased abundance of beneficial taxa, including *Alistipes*, *Akkermansia*, *[Eubacterium]_siraeum*_group, and *Intestinimonas* (Figure 7H–L). Linear discriminant analysis effect size (LEfSe) analysis revealed *Verrucomicrobia* (phylum), *Rikenellaceae*, *Akkermansiaceae*, as dominant in WT mice, whereas *DR5* ^−/−^ mice exhibited enrichment of *Tannerellaceae* (Figure 7M), a family of *Bacteroidetes* that secretes endotoxins that may accelerate disease progression.^[^ ^34^ ^]^ qPCR revealed a significant reduction in *Akkermansia muciniphila* (*A. muciniphila*) in *DR5* ^−/−^ mice (Figure 7N), consistent with the mucus layer deficiency, as *A. muciniphila* relies on mucin O‐glycans as the nutrient source.^[^ ^35^ ^]^ However, despite increasing mucus layer thickness, a 7‐day Bioymifi enema treatment did not alter *A. muciniphila* abundance, possibly due to the short time frame for microbial community restructuring (Figure S8, Supporting Information). The colon tissue of *DR5* ^−/−^ mice exhibited significantly higher mRNA expression of pro‐inflammatory cytokines (*Tnf‐α*, *Il‐1β*, *Il‐6*) compared to WT mice, but did not develop significant histopathological inflammation or epithelial damage under homeostatic conditions (Figure S9, Supporting Information). These data delineate that *DR5* knockout alters microbial ecology toward pro‐inflammatory taxa while impairing mucus‐dependent commensal colonization due to mucus layer deficiency.

### 2.7 *DR5* Knockout Reduces the Resistance of the Mice to *Citrobacter rodentium* Infection

**Figure 8:**
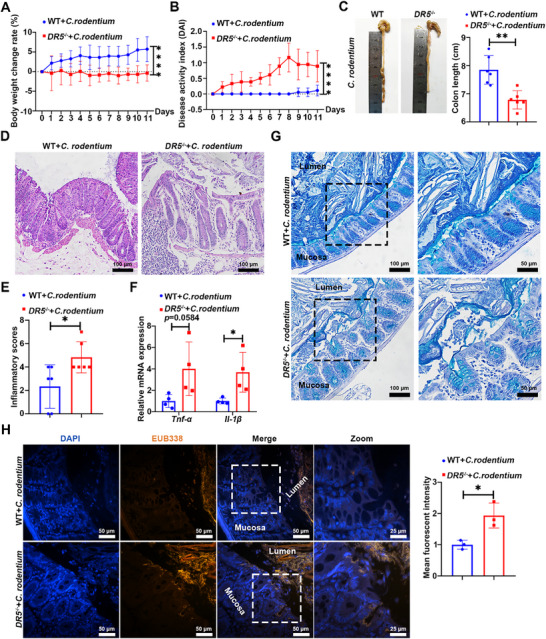
*DR5* knockout impairs host defense against *C. rodentium* infection. WT and *DR5* ^−/−^ mice were orally gavaged with 10^9^ CFU mL^−1^ *C. rodentium* (200 µL per mouse) to induce colonic inflammation and sacrificed at day 11 post‐infection. A) Daily body weight change rate following *C. rodentium* infection in WT and *DR5* ^−/−^ mice (*n* = 6). B) Daily disease activity index following *C. rodentium* infection in WT and *DR5* ^−/−^ mice (*n* = 6). C) Quantification of colon length in WT and *DR5* ^−/−^ mice at day 11 post‐infection (*n* = 6). D) Representative images of Hematoxylin and eosin (H&E)‐stained colon sections in WT and *DR5* ^−/−^ mice at day 11 post‐infection. E) Inflammatory scores of colon tissues from WT and *DR5* ^−/−^ mice at day 11 post‐*C. rodentium* infection, based on H&E‐stained colon sections (*n* = 6). F) qRT‐PCR analysis of the inflammatory cytokine mRNA expression in the colon of WT and *DR5* ^−/−^ mice at day 11 post‐*C. rodentium* infection (*n* = 4). G) Images of AB staining showing mucus layer and mucin in WT and *DR5* ^−/−^ mice at day 11 post‐*C. rodentium* infection. H) Representative images for bacterial colonization in the colon detected by the bacterial EUB338 probe at day 11 after *C. rodentium* infection. Epithelial‐adherent bacteria were quantified based on EUB338 mean fluorescence intensity (10 regions per mouse; *n* = 3). Data are expressed as mean ± SD. Data in Figure 8A,B were analyzed by two‐way ANOVA followed by Sidak's post hoc test. Other data were analyzed by an unpaired *t*‐test. ^*^ *p* <0.05, ^**^ *p* <0.01, ^****^ *p* <0.0001.

Given that mucus barrier dysfunction promotes pathogen penetration and host susceptibility,^[^ ^36^ ^]^ we challenged mice with *Citrobacter rodentium* (*C. rodentium*). Compared to WT mice, infected *DR5* ^−/−^ mice showed increased weight loss, elevated disease activity index, and reduced colon length (**Figure** **8**A–C). Histopathological analysis at day 11 post‐infection revealed enhanced inflammatory cell infiltration with upregulated *Tnf‐α*, *Il‐1β* expression in *DR5* ^−/−^ mouse colons vs WT control (Figure 8D–F). Critically, AB staining and 16S rDNA FISH revealed closer bacterial proximity to the epithelium and increased bacterial adhesion in *DR5* ^−/−^ mice, demonstrating enhanced invasion (Figure 8G,H). These findings establish that DR5 mediates critical barrier defense by preserving mucus integrity to prevent pathogen translocation.

## 3 Discussion

The mucus layer secreted by intestinal goblet cells constitutes a critical interface that orchestrates host‐microbiota interactions and safeguards epithelial integrity.^[^ ^37^ ^]^ While its functional significance is well‐established, the molecular regulation of goblet cell secretion remains incompletely defined. Here, we identify DR5 in goblet cells as a critical regulator of colonic mucus layer homeostasis through a previously unrecognized, non‐apoptotic effect: governing goblet cell compound exocytosis. *DR5* knockout results in a mild colonic dysbiosis under steady‐state, rendering mice susceptible to *C. rodentium* invasion and exacerbating epithelial injury. Mechanistically, DR5 regulates compound exocytosis in goblet cells through TBP‐mediated transcription of *Best2*, a colonic goblet cell‐specific HCO_3_^−^ channel. Our findings delineate a novel DR5‐TBP‐Best2 signaling axis in colonic goblet cells, which is crucial for governing mucus exocytosis and barrier defense.

DR5 is a membrane receptor with dual and opposing functions: it triggers apoptosis upon binding TRAIL at the plasma membrane, yet promotes survival and/or proliferation following nuclear translocation.^[^ ^17^ ^]^ Beyond its canonical membrane localization, we identified DR5 in the cytosol and nucleus of colonic goblet cells. In the intestine, the mucus layer undergoes continuous degradation by microbial enzymes and mechanical shedding by peristalsis; its thickness is maintained through ongoing mucin synthesis and secretion by goblet cells.^[^ ^12^, ^37^, ^38^ ^]^ Muc2, the primary gel‐forming mucin, serves as the structural scaffold of this layer.^[^ ^39^ ^]^ Our central finding—the coexistence of a thin mucus layer with unchanged expression of goblet cell differentiation/maturation marker, elevated Muc2 protein level, intracellular mucin hyper‐accumulation with normal glycosylation—strongly suggests that impaired mucin secretion, rather than defective synthesis underlies the mucus layer defect in *DR5* ^−/−^ mice. To test this, we employed CCh to trigger compound exocytosis—a process essential for high‐volume mucus release and barrier renewal.^[^ ^23^, ^40^, ^41^ ^]^ Unlike in WT colonic organoids, CCh failed to elicit Muc2 releasing in *DR5* ^−/−^ organoids. Consistent with a functional TRAIL/DR5 axis, *TRAIL* knockdown or TRAIL mimetic Bioymifi administration^[^ ^24^ ^]^ respectively impaired or enhanced CCh‐induced Muc2 exocytosis in organoids. Building on the evidence from colonic organoids that epithelial DR5 regulates compound exocytosis, we confirmed that pharmacological DR5 activation with Bioymifi increases mucus layer thickness in vivo. This key finding strongly supports the translational potential of modulating this pathway for therapeutic benefit. As DR5 deficiency did not detectably alter epithelial apoptosis in the colon, the observed phenotypes are independent of its classical pro‐death function. Collectively, these results define a previously unrecognized, non‐apoptotic function for the TRAIL/DR5 axis in regulating goblet cell compound exocytosis under homeostasis.

Muc2 polymers are densely packed within secretory vesicles and shielded by cations, primarily Ca^2+^ and H^+^.^[^ ^9^ ^]^ In this process, HCO_3_^−^ plays a critical role: it facilitates mucin exocytosis and subsequent expansion by chelating Ca^2^⁺ and elevating intra‐granular pH.^[^ ^42^, ^43^ ^]^ Data from CCD 841 CoN cells suggest that DR5 modulates goblet cell compound exocytosis by establishing an optimal intracellular pH. This is supported by the finding that *DR5* knockdown elevated intracellular acidity—a condition suppressing secretory granule release,^[^ ^25^ ^]^ aligning with impaired Muc2 secretion in *DR5* ^−/−^ organoids. Conversely, Bioymifi promoted intracellular alkalization, a prerequisite for Muc2 granule depolymerization and release,^[^ ^9^ ^]^ consistent with its ability to enhance compound exocytosis. The colon‐specific nature of DR5's effect, as well as the pH sensitivity of granule exocytosis suggests the involvement of a colon‐restricted pH regulatory mechanism. Best2, a basolateral HCO_3_^−^ channel exclusively expressed in human and mouse colonic—not small intestinal—goblet cells represents a compelling candidate mediator.^[^ ^26^, ^27^ ^]^ DR5 consistently regulated Best2 expression, with knockout downregulating and activation upregulating Best2 at both transcriptional and protein levels in colonic goblet cells. The Best2 channel creates a directional HCO_3_^−^ flow by mediating basolateral uptake and apical efflux in the colon.^[^ ^26^ ^]^ While technical limitations restricted direct intracellular pH measurements in primary goblet cells, we assessed apical pH from colonic explants as a proxy. Functionally, *DR5* knockout impaired CCh‐induced HCO_3_^−^ secretion in colonic explants, while DR5 activation enhanced it. This concordance between DR5‐dependent HCO_3_^−^ secretion and Best2 expression supports a model in which DR5 regulates basolateral HCO_3_^−^ influx via Best2, leading to HCO_3_^−^ accumulation within colonic goblet cells that facilitates mucin release and expansion.^[^ ^10^, ^26^, ^43^ ^]^ The essential role of Best2 was further confirmed by knockdown experiments in colonic organoids, which not only inhibited CCh‐induced Muc2 secretion but also abrogated the stimulatory effect of DR5 activation on it. The co‐expression of DR5, TRAIL, and Best2 within crypt goblet cells—the primary site for compound exocytosis^[^ ^44^, ^45^ ^]^—also supports a functional DR5‐Best2 signaling axis. Beyond compound exocytosis, basal secretion is also crucial for maintaining the steady‐state mucus barrier.^[^ ^46^ ^]^ We found that *DR5* knockout also inhibited basal secretion, but through a mechanism distinct from that governing compound exocytosis, as *Best2* interference did not affect basal secretion in organoids (unpublished data). Thus, the basis for DR5's control of basal secretion therefore warrants separate investigation.

To elucidate how DR5 regulates Best2 expression, we established *DR5*‐knockout and *DR5*/*TRAIL*‐co‐overexpressing cell models. Consistent with in vivo observations, the results demonstrate that DR5 transcriptionally regulates *BEST2* through a ligand‐dependent mechanism requiring functional DD, aligning with the canonical requirement for ligand‐induced oligomerization in death receptor signaling.^[^ ^47^ ^]^ However, DR5 did not bind the *BEST2* promoter directly, as shown by ChIP‐qPCR, indicating an indirect mechanism. Integrated bioinformatic analysis and co‐IP of the DR5 interactome, together with observed DR5‐TBP physical interaction, suggested TBP as a potential intermediary. This hypothesis was confirmed by TBP's direct binding to the *BEST2* promoter and its ability to enhance *BEST2* transcription and promoter activity. Furthermore, DR5 signaling consistently influenced the expression of TBP and Best2 in parallel. Critically, *TBP* knockdown in organoids abolished DR5 activation‐induced upregulation of *Best2* mRNA and the associated enhancement of goblet cell compound exocytosis, establishing the DR5‐TBP‐Best2 axis as essential for goblet cell secretion. While our study establishes DR5‐mediated upregulation of TBP, the precise mechanistic link remains an important area for future investigation. It is plausible that DR5 activation initiates kinase pathways, such as NF‐κB, MAPKs (ERK1/2, JNK), PI3K/Akt,^[^ ^48^, ^49^ ^]^ which may directly or through intermediate TFs orchestrate *TBP* transcription. In addition, the nuclear co‐localization of DR5 and TBP suggests a nuclear paradigm. Nuclear‐localized DR5 might exert direct transcriptional control over the *TBP* promoter, akin to its role in modulating microRNA maturation and gene expression in other contexts.^[^ ^17^, ^50^, ^51^ ^]^ Alternatively, the DR5‐TBP interaction may not be the cause of the increased TBP expression, but rather can regulate the post‐translational activity of TBP, for instance, by stabilizing the TBP protein or promoting its specific recruitment to the *Best2* promoter. Future work dissecting these possibilities will be essential to fully unravel this regulatory axis. In summary, our data establish that DR5 controls *Best2* transcription by modulating TBP expression, which functions as the TF directly activating the *Best2* promoter.

The gut microbiota composition is critically influenced by the mucus layer, which provides a habitat for commensal bacteria.^[^ ^52^, ^53^ ^]^ *DR5* ^−/−^ mice exhibits microbiota dysbiosis characterized by a functional shift toward pathogen susceptibility and reduced mucosal protection, indicating an elevated risk of colonic inflammation. We acknowledge the sample size for 16S sequencing was relatively modest, so we further validated the expression of *A. muciniphila* to enhance this finding. The observed dysbiosis, particularly the decline in *A. muciniphila*—a mucus‐dependent symbiont^[^ ^35^ ^]^—directly correlates with impaired mucus barrier integrity in *DR5* ^−/−^ mice. Although our microbiome analysis confirmed the physiological consequence of an impaired mucus layer, a deeper mechanistic understanding of how DR5‐dependent mucus changes shape the microbial community will require future integration of metagenomic or metabolomic approaches. Despite this dysbiosis, no overt inflammatory phenotype is observed in the colon under homeostasis, aside from elevated pro‐inflammatory cytokines. There may be other epithelial barrier mechanisms that compensate for the insufficiency of the mucus layer, such as the epithelial mechanical barrier. Nevertheless, mucus barrier impairment increased host susceptibility to the attaching/effacing pathogen *C. rodentium* ^[^ ^54^ ^]^ by enhancing adhesion of bacteria to the intestinal epithelium. TRAIL/DR5 system participates in inflammatory bowel disease, while current evidence remains conflicting, and the underlying mechanisms are not fully elucidated. Consistent with our findings, previous studies confirmed TRAIL/DR5's protective role in colonic inflammation: intraperitoneal recombinant soluble TRAIL reduced colitis severity in mice by inhibiting colitogenic T‐cell activation via DR5 activation;^[^ ^55^ ^]^ whereas *TRAIL* ^−/−^ and *DR5* ^−/−^ mice exhibited exacerbated DSS‐induced colitis independent of apoptosis.^[^ ^22^, ^56^ ^]^ Our data suggest the mucus barrier as a mechanistic contributor. Conversely, a study reported that sDR5‐Fc ameliorated colitis symptoms by competitively inhibiting TRAIL‐DR5 binding in mice.^[^ ^57^ ^]^ These discrepancies may reflect cell type‐specific functions of DR5, warranting targeted investigation into TRAIL/DR5's cell type‐specific effects.

In summary, our findings establish a novel, non‐canonical role for DR5 in regulating goblet cell compound exocytosis and mucus layer homeostasis by transcriptionally regulating *Best2* in colonic goblet cells. The DR5‐TBP‐Best2 axis represents a novel regulatory pathway, positioning the precision targeting of goblet cell DR5 signaling as a promising therapeutic strategy for reinforcing the mucus barrier in colitis. This study focused on the mechanism of compound exocytosis; consequently, the role of DR5 in basal secretion and the mechanism governing TBP expression merit further investigation.

## 4 Experimental Section

### Animals

Male C57BL/6N mice (6–8 weeks, 20–22 g) were obtained from Beijing Vital River Laboratory Animal Technology Co., Ltd. *DR5* heterozygous (*DR5* ^+/−^) mice on a C57BL/6N genetic background were purchased from Saiye Suzhou Biological Technology Co., Ltd., China, and *DR5* knockout (*DR5* ^−/−^) mice aged 6–8 weeks were used in this study. All mice were housed in a temperature‐controlled environment (22—26 °C) under a 12/12‐h light/dark cycle with ad libitum access to food and water. All animal experiments were approved by the Animal Ethics and Welfare Committee, School of Basic Medicine, Shandong University (Shandong, China).

### Establishment of *C. rodentium* Infection Model

*C. rodentium* infection model was established as previously described.^[^ ^58^ ^]^ Briefly, the *C. rodentium* strain DBS100 (ATCC) was cultured overnight in Luria‐Bertani (LB) medium (Beijing Solarbio Science & Technology Co., Ltd., China) at 37 °C. The next day, bacteria were harvested and resuspended in PBS. Mice were administered 200 µL of *C. rodentium* suspension (10⁹ CFU mL^−1^) by oral gavage to induce intestinal infection. The disease activity index, including body weight loss, stool consistency, and fecal bleeding, was monitored daily following the literature.^[^ ^59^ ^]^ Mice were euthanized at day 11 post‐infection, and colons were collected, measured for length, and then either fixed or frozen for subsequent analysis.

### Hematoxylin and Eosin (H&E) Staining, Periodic Acid–Schiff (PAS) Staining, and High Iron Diamine/Alcian Blue (HID/AB) Staining

Distal colon tissues were fixed in 4% paraformaldehyde for 48 h, dehydrated through a graded ethanol series, embedded in paraffin, and sectioned into 4‐µm‐thick slices for H&E, PAS, or HID/AB staining. Intestinal inflammation was assessed with a published colitis activity index^[^ ^59^ ^]^ based on blinded analysis of H&E‐stained sections. The index evaluated four histopathological parameters: inflammation severity and extent, crypt damage, and percentage involvement. Each parameter was graded from 0 (absent) to 3 or 4 (severe), yielding a maximum total score of 14 indicating the most severe tissue damage.^[^ ^59^ ^]^ PAS staining was performed using the PAS kit (Fuzhou Maixin Biotech. Co., Ltd., China) according to the manufacturer's instructions to detect neutral mucins in goblet cells. The mucin area per goblet cell was quantified by ImageJ software (Bio‐Rad, Hercules, USA). For mucin area quantification, the average value from at least 10 randomly selected crypts per mouse was calculated. Mucin quality was assessed by histochemical staining using an HID/AB kit (Shanghai yuanye Bio‐Technology Co., Ltd., China) per the manufacturer's protocol. This method differentially stains sialomucins (blue, AB‐positive) and sulfomucins (brown, HID‐positive).^[^ ^60^ ^]^ The sulfomucin‐to‐sialomucin ratio in colonic goblet cells was quantified with ImageJ software by analyzing five randomly selected fields (200 × magnification) per mouse. All assessments were blinded and performed by two independent investigators.

### Terminal Deoxynucleotidyl Transferase‐Mediated dUTP Nick‐End Labeling (TUNEL) Assay

IEC apoptosis was assessed using a TUNEL Apoptosis Assay Kit (Keygen Biotech, China). Briefly, paraffin‐embedded sections were baked, dewaxed, and rehydrated. After PBS washing, the sections were digested with proteinase K for 30 min and incubated with the TdT enzyme reaction solution at 37 °C for 1 h. This was followed by a 30 min incubation with streptavidin‐fluorescein at 37 °C. Nuclei were counterstained with DAPI, and the slides were visualized under a fluorescence microscope (Nikon, Japan).

### 16S rRNA Gene Amplicon Sequencing

Fecal microbiome analysis was performed through 16S rRNA gene amplicon sequencing. DNA samples were amplified with barcode primers 515F (5′‐GTGCCAGCMGCCGCGGTAA‐3′) and 806R (5′‐GGACTACHVGGGTWTCTAAT‐3′), targeting the V4 region of the bacterial 16S rRNA gene, and were sequenced on the Illumina NovaSeq 6000. Alpha diversity (Chao1, Simpson, and Shannon indices), beta diversity (Bray‐Curtis and Jaccard distances), species composition, and LEfSe were analyzed using the NovoMagic platform (Novogene Co., Ltd., Beijing, China) to characterize the gut microbiota structure.

### qPCR Analysis of *A. muciniphila* Relative Abundance in Fecal Samples

Stool microbial DNA was extracted using the TIANamp Stool DNA Kit (Tiangen Biotech Co., Ltd., Beijing, China) according to the manufacturer's instructions. qPCR was performed using UltraSYBR Mixture (Beijing Cowin Biotech Co., Ltd., China) on an Applied Biosystems StepOne Real‐Time PCR System (Thermo Fisher Scientific, USA). The relative abundance of *A. muciniphila* in stool was normalized to the 16S rRNA gene level of total bacteria. qPCR relative quantification was performed with the following primers: forward: 5′‐TCCTACGGGAGGCAGCAGT‐3′ and reverse: 5′‐GGACTACCAGGGTATCTATCCTGTT‐3′ for total bacteria; forward: 5′‐GACCGGCATGTTCAAGCAGACT‐3′ and reverse: 5′‐AAGCCGCATTGGGATTATTTGTT‐3′ for *A. muciniphila*.

### Immunohistochemistry and Immunofluorescence

For immunohistochemistry, tissue slides were deparaffinized in xylene, rehydrated through a graded alcohol series, and subjected to heat‐induced antigen retrieval in 0.01 m citrate buffer (pH 6.0). Endogenous peroxidase activity was blocked by incubation with the peroxidase blocker for 30 min. Sections were then incubated with primary antibodies overnight at 4 °C. Immunodetection was performed using a two‐step polymer detection kit (Beijing Zhongshan JQ Biotechnology Co., Ltd., China). Nuclei were counterstained with hematoxylin (Solarbio).

For immunofluorescence, following deparaffinization, rehydration, and antigen retrieval (as described for immunohistochemistry), sections were incubated with 3% H_2_O_2_ at room temperature for 30 min to quench endogenous peroxidase activity. Sections were permeabilized with 0.5% Triton‐X‐100 for 10 min, and blocked with goat serum (Solarbio) at 37 °C for 1 h. Primary antibodies were applied to sections overnight at 4 °C. Following washes with PBS, sections were incubated with appropriate secondary antibodies in a humidified chamber for 1 h in the dark at 37 °C. Nuclei were stained with 4,6‐diamidino‐2‐phenylindole (DAPI; Shanghai Beyotime Biotechnology Co., Ltd., China) and images were acquired via a fluorescence microscope (Nikon, Japan).

All antibodies used were listed in Table S2 (Supporting Information).

### Transmission Electron Microscopy

Colonic segments were removed, flushed, and minced into ≈1 mm^3^ tissue blocks. Then, the blocks were immediately fixed in 2.5% glutaraldehyde in 0.1 m sodium cacodylate for 4 h followed by 1% osmium tetroxide in 0.1 m phosphate buffer for 1 h at 4 °C. Subsequently, samples were dehydrated through a graded ethanol series, infiltrated, and embedded in epoxy resin. Ultrathin sections (70 nm) were stained with uranyl acetate and lead citrate, and imaged using a JEM‐1200EX transmission electron microscope (Hitachi Electronic Company, Japan).

### Alcian Blue (AB) Staining and 16S rDNA FISH

Distal colon segments containing luminal feces were immediately fixed in Carnoy's solution to preserve the mucus layer, embedded in paraffin, and sectioned (4 µm) for AB staining or 16S rDNA FISH. After deparaffinization and rehydration, the paraffin‐embedded sections were incubated in AB solution (pH 2.5; Zhuhai Baso Biotechnology Co., Ltd., China) for 15 min at room temperature. Nuclei were counterstained with hematoxylin. 16S rDNA FISH was performed using a commercial FISH kit (Wuhan Servicebio Technology, Co., Ltd., China) according to the manufacturer's instructions. Briefly, following deparaffinization and rehydration, sections were subjected to heat‐induced antigen retrieval at 90 °C for 10 min. Then, sections were digested with Protease K at 37 °C for 30 min, followed by three PBS washes to terminate enzymatic activity. Sections were hybridized overnight at 37 °C with Alexa Fluor 555‐conjugated universal bacterial probe EUB338 (5′‐GCTGCCTCCCGTAGGAGT‐3′; Servicebio) in a humidified chamber. After stringent washes with 2 × saline sodium citrate (SSC) buffer at 37 °C and counterstaining with DAPI, images were acquired using a fluorescence microscope to quantify the thickness of the inner mucus layer in the distal colon. For each mouse, the thickness of the inner mucus layer was measured in at least 10 randomly selected fields (200 × magnification) to calculate the mean value. All measurements were performed blindly by two independent investigators.

### Intracolonic Infusion of Bioymifi

Mice received daily intracolonic infusions of 200 µL DR5 agonist Bioymifi (100 nm) or vehicle control for 7 days using a flexible plastic tube.^[^ ^61^ ^]^ Animals were euthanized 24 h after the final infusion. The distal colon segments were harvested for ex vivo pH measurement, and fixed in Carnoy's solution (with luminal feces preserved) or fixed in paraformaldehyde (after fecal content removal) for PAS staining.

### Colonic Organoid Culture and Experimental Protocol

Colonic crypts were isolated as previously described.^[^ ^62^ ^]^ Briefly, distal colon segments from WT mice or *DR5* ^−/−^ mice were longitudinally opened, rinsed with ice‐cold DPBS, and cut into 2–3 mm fragments, followed by repeated washing in cold DPBS. Crypts were isolated by incubation with 2 mm EDTA (Invitrogen, USA) in a shaker at 4 °C for 1 h, collected by centrifugation at 300 × g for 5 min at 4 °C, and quantified microscopically. A total of 150 crypts were resuspended in 50 µL Matrigel (Corning Inc., USA) and seeded into 48‐well plates. After Matrigel solidification, 250 µL Mouse Colonic Organoid Medium (bioGenous BIOTECH, Inc., China) was added to each well, and crypts were cultured at 37 °C in a humidified air containing 5% CO_2_. Medium was replaced every 3 days. To selectively activate DR5, 3‐day‐cultured organoids were treated with the small molecule compound Bioymifi (100 nm)^[^ ^20^ ^]^ for 48 h. For gene silencing, 5‐day‐cultured organoids were dissociated in Cell Recovery Solution (Corning), digested to single cells with trypsin‐EDTA, and transfected for 4 h with siRNA (1 nmol, Shanghai GenePharma Co., Ltd., China) complexed with 3 µL lipofectamine3000 (Invitrogen, USA) in 200 µL Opti‐MEM medium at 37 °C in a humidified air containing 5% CO_2_. Three distinct siRNAs targeting genes *TRAIL* (si‐*TRAIL*), *Best2* (si‐*Best2*), and *TBP* (si‐*TBP*) were used (Table S3, Supporting Information), with a non‐targeting siRNA as a negative control (NC). The transfected cells were resuspended in Matrigel and reseeded in 48‐well plates. After the Matrigel solidified, Mouse Colonic Organoid Medium with or without Bioymifi (100 nm) was added, followed by 48 h of culture.

### Dot Blotting to Evaluate Goblet Cell Compound Exocytosis

The organoids were stimulated with CCh (100 µm) to induce compound exocytosis in goblet cells.^[^ ^23^ ^]^ Muc2 content in colonic organoid supernatants was quantified by dot blotting^[^ ^63^ ^]^ to assess goblet cell compound exocytosis. The supernatants were spotted on nitrocellulose membranes (Millipore, Massachusetts, USA) and cross‐linked at 37 °C for 30 min. Membranes were blocked with 4% bovine serum albumin (BSA; Beijing Seaskybio Technology Co., Ltd., China) for 1 h at room temperature, followed by overnight incubation at 4 °C with anti‐Muc2 antibody (1:1000; Santa Cruz Biotechnology). After washing, membranes were incubated with an HRP‐conjugated Goat Anti‐Mouse IgG (H+L) secondary antibody (1:5000; Proteintech) for 1 h at room temperature. Signals were detected using enhanced chemiluminescence blotting reagents (Beyotime) and quantified using ImageJ software by densitometry.

### Cell Lines and Culture Conditions

The human normal colon epithelial cell line CCD 841 CoN (Research Resource Identifiers, RRID: CVCL_2871) was acquired from ATCC. The human embryonic kidney cell line HEK293T (RRID: CVCL_0063) was purchased from Wuxi Puhe Biotechnology Co., Ltd., China. Cell lines were confirmed to be free of mycoplasma contamination. All cells were cultured in Dulbecco's Modified Eagle's Medium (DMEM; Shanghai BasalMedia Technology Co., Ltd., China) supplemented with 10% fetal bovine serum (Suzhou Shuangru Biotech Co., Ltd., China), 100 U mL^−1^ penicillin, and 0.1 mg mL^−1^ streptomycin (Solarbio). Cultures were incubated at 37 °C in a humidified incubator containing 5% CO_2_.

### Plasmids and siRNAs Transfection

Plasmids encoding GFP‐*BEST2*, Flag‐*TBP*, wild‐type Flag‐*DR5* (Flag‐*DR5* WT), or its death domain (DD)‐inactive mutant (Flag‐*DR5* Mut) were cloned into the pcDNA3.1 vector. The His‐*TRAIL* plasmid was constructed in the PEnter vector. All plasmids were purchased from Boshang Biotechnology Co., Ltd., China. Gene‐specific siRNAs targeting *DR5*, *TRAIL*, *Best2*, *TBP* were utilized and purchased from Shanghai GenePharma Co., Ltd., China. Transient transfection of plasmids or siRNAs was performed using lipofectamine3000 following the manufacturer's instructions. Briefly, cells were seeded in complete medium. Upon reaching 70% confluence, they were transfected with either plasmids or siRNAs using lipofectamine3000 for 6 h. Following transfection, the medium was replaced with fresh complete medium, and cells were cultured for another 24 or 48 h for subsequent experiments.

### The Intracellular pH Detection in CCD 841 CoN Cells

CCD 841 CoN cells were seeded in 24‐well plates and subjected to the following treatments: transfection with *BEST2* plasmid (0.8 µg) or empty vector; transfection with si‐*DR5* (20 pmol) or NC; treatment with Bioymifi (100 nm) or vehicle. 48 h post‐transfection or Bioymifi treatment, cells were incubated with 1 µm BCECF AM (Beyotime) for 30 min at 37 °C.^[^ ^64^ ^]^ After staining, cells were washed three times with PBS and imaged using a fluorescence microscope. The higher the fluorescence intensity, the stronger the alkalinity, and vice versa.

### Measurement of Supernatant pH in Colonic Explants

The pH in the supernatant of colonic explants was measured following a previously described method for assessing intestinal lumen pH in vivo, with modifications.^[^ ^65^ ^]^ Briefly, a 10 mg segment of the distal colon was rapidly excised and immersed in 80 µL of ultrapure water in a microcentrifuge tube. CCh was immediately added to a final concentration of 1 mm. After 10 min of incubation, the pH of the supernatant was determined using a pH meter (METTLER TOLEDO, Shanghai, China) to assess the CCh‐induced apical HCO_3_^−^ secretion.

### Dual‐Luciferase Reporter Assay

The human *BEST2* promoter was cloned into the pGL3‐Basic luciferase reporter vector (Boshang Biotechnology). CCD 841 CoN cells were co‐transfected with pRenilla‐TK, pGL3‐*BEST2* or empty pGL3 vector, si‐*DR5* or NC. In parallel, HEK293T cells were co‐transfected with pRenilla‐TK, pGL3‐*BEST2* or pGL3 vector, and *DR5* WT/Mut and *TRAIL* plasmids or their respective vector controls. At 48 h post‐transfection, luciferase activity was detected using the Dual‐Luciferase Reporter Assay System (Promega Corporation, Madison, WI, USA) with a dual luciferase reporter gene assay kit (Vazyme Biotech Co., Ltd., Wuhan, China). Firefly luciferase activity values were normalized to Renilla luminescence and expressed as relative luciferase activity.

### Prediction of the Transcription Factors for BEST2

To identify TFs regulating *BEST2* transcription, its promoter region (2000 bp upstream of the transcription start site, TSS) was retrieved from the National Center for Biotechnology Information Gene. This sequence was analyzed using three independent TF prediction databases: JASPAR, GTRD, and HumanTFDB. Common TFs identified across all databases were determined through comparative Venn diagram analysis.

### Liquid Chromatography‐Tandem Mass Spectrometry (LC‐MS/MS) Analysis and Functional Enrichment Analysis

CCD 841 CoN cells were lysed in Pierce IP lysis buffer (Thermo Fisher Scientific, USA) on ice for 30 min, followed by centrifugation (13400 × g, 15 min, 4 °C). Supernatants were immunoprecipitated with anti‐DR5 antibody (1:50; Abcam) for 4 h at 4 °C, then incubated with Protein A/G Agarose (Wuhan Dia‐An Biotechnology Co., Ltd., China) overnight at 4 °C. Precipitated complexes were digested for LC‐MS/MS analysis (Shanghai Genechem Co., Ltd., China). Gene Ontology enrichment analysis for the MS results was carried out by using the *R* ClusterProfiler package. After converting the IDs of the input molecule list, enrichment analysis was conducted with the ClusterProfiler package, and the top 10 pathways in biological (BP), cellular components (CC), and molecular functions (MF) were visualized. The threshold for statistical significance in the MS analysis was defined as *p*.adj <0.05.

### Co‐IP in Colonic Crypt Cells

Mouse colonic crypt cells were isolated and processed identically. Lysates were immunoprecipitated with anti‐DR5 (1:50, Abcam), anti‐TBP (1:20, Santa Cruz), or anti‐IgG (1:100, Proteintech), followed by incubation with protein A/G beads to bind the immune complexes. After extensive washing, immunoprecipitated proteins were detected by western blot.

### ChIP‐qPCR

ChIP assays were performed in HEK293T cells using a commercial ChIP kit (Cell Signaling Technology, USA) according to the manufacturer's instructions. To examine DR5 and TBP binding to the *BEST2* promoter, HEK293T cells were transfected with either Flag‐*DR5* WT overexpression plasmid (for DR5 binding analysis) or Flag‐*TBP* overexpression plasmid (for TBP binding analysis) for 6 h. Following transfection, the medium was replaced with fresh medium containing 100 nm Bioymifi (for Flag‐*DR5*‐transfected cells) or without Bioymifi (for Flag‐*TBP*‐transfected cells). Cells were then cultured for an additional 72 h. After treatment, cells were cross‐linked with 1% formaldehyde (Sigma–Aldrich, USA) for 15 min at room temperature. The reaction was quenched with glycine, and chromatin was isolated and sonicated to yield fragments of 200‐500 bp DNA fragments. Chromatin lysates were immunoprecipitated overnight at 4 °C with anti‐Flag (1:50; Proteintech) and anti‐IgG antibody (1:100; Cell Signaling Technology). Immunoprecipitated DNA was analyzed by qPCR. Specific genomic regions within the *BEST2* promoter were amplified using the following primers: for the DR5‐bound region, forward: 5′‐ACCAGACAGGAAACCGCAAG‐3′ and reverse: 5′‐ACACACTTCCGACTGCTGAA‐3′; for the TBP‐bound region: forward: 5′‐ CATGACGGAATTCAGACCGTT ‐3′ and reverse: 5′‐ GGGTCACACAAGACACACCTA ‐3′.

### Western Blot

Total protein was extracted from colon tissues, colonic organoids, or cells. Protein concentrations were quantified using a Bicinchoninic Acid Protein Assay kit (Beyotime). Proteins were separated by SDS‐PAGE, transferred to polyvinylidene difluoride membrane (Millipore, Massachusetts, USA), and blocked with 5% skim milk for 2 h at room temperature. Membranes were incubated overnight at 4 °C with primary antibodies: anti‐Muc2 (1:1000; Abcam), anti‐TBP (1:1000; Santa Cruz), anti‐DR5 (1:1000; Abcam), anti‐Cleaved caspase‐3 (1:1000; Cell Signaling Technology), anti‐Caspase‐3 (1:1000; Proteintech). The next day, membranes were washed with TBST and incubated with HRP‐conjugated Goat Anti‐Rabbit IgG(H+L) (1:5000; Proteintech) or HRP‐conjugated Goat Anti‐Mouse IgG(H+L) (1:5000; Proteintech) for 1 h. Protein bands were visualized on a ChemiDoc XRS system using BeyoECL PLUS (Beyotime) and quantified with ImageJ software.

### mRNA Isolation and qRT‐PCR

Total RNA was isolated from colon tissues, organoids, or cells using SparkZol Reagent (Shandong Sikejie Biotechnology Co., Ltd., China). cDNA was reverse‐transcribed using PrimeScript RT Reagent Kit (Takara, Japan) following the manufacturer's instructions. qPCR was conducted using UltraSYBR Mixture (Cowin) on an Applied Biosystems StepOne Real‐Time PCR System. Gene expression levels were normalized to *β‐actin or GAPDH* and quantified using the 2^−ΔΔCt^ method. Primer sequences are provided in Table S4 (Supporting Information).

### Drugs and Chemicals

The selective DR5 agonist Bioymifi was purchased from Selleck Chemicals (Houston, TX, USA). It was prepared as a 10 mm stock solution in dimethyl sulfoxide (DMSO) and diluted to a final working concentration of 100 nm for all the experiments. CCh was obtained from Shanghai Yien Chemical Technology Co., Ltd., China.

### Statistical Analyses

Data are presented as mean ± SD. Statistical analyses were performed using GraphPad Prism 8.0 (San Diego, CA, USA). Normality was assessed with the Shapiro–Wilk test. Normally distributed data were analyzed by unpaired *t*‐tests, while non‐normally distributed data were compared using Mann–Whitney U test for two‐group comparisons. Multi‐group comparisons were analyzed using One‐way ANOVA followed by Tukey's post hoc test or two‐way ANOVA followed by Sidak's post hoc test. The sample size “n” for each experiment is defined in the figure legends and represents the number of biological replicates. For animal studies, “n” indicates the number of individual mice. For organoid experiments, “n” indicates the number of independent biological replicates, each derived from a separate primary crypt isolation. For cell line experiments, “n” represents the number of independent experiments performed with cells cultured in distinct passages. Statistical significance was defined as *p* <0.05.

### Ethics Approval

All procedures were conducted in compliance with relevant guidelines and regulations, and all animal experiments were approved by the Animal Care and Use Committee of the School of Basic Medical Science, Shandong University.

## Conflict of Interest

The authors had no conflict of interest.

## Author Contributions

Y.W. performed the experiments, acquired and analyzed the data. X.L., Y.W., and C.Y. helped to perform ex vivo experiments. X.G., K.Z., and Y.R. collected the histological data. J.L. and C.L. contributed to the experimental design. B.X. designed the study, wrote the manuscript, and provided financial support.

## Supporting information



Supporting Information

## Data Availability

The data that support the findings of this study are available from the corresponding author upon reasonable request.

## References

[1] M. E. Johansson , G. C. Hansson , Nat. Rev. Immunol. 2016, 16, 639. 27498766 10.1038/nri.2016.88 PMC6435297

[2] M. E. Johansson , J. M. Larsson , G. C. Hansson , Proc. Natl. Acad. Sci. USA 2011, 108, 4659.20615996

[3] S. van der Post , K. S. Jabbar , G. Birchenough , L. Arike , N. Akhtar , H. Sjovall , M. E. V. Johansson , G. C. Hansson , Gut 2019, 68, 2142. 30914450 10.1136/gutjnl-2018-317571 PMC6872445

[4] M. E. V. Johansson , J. K. Gustafsson , J. Holmén‐Larsson , K. S. Jabbar , L. Xia , H. Xu , F. K. Ghishan , F. A. Carvalho , A. T. Gewirtz , H. Sjövall , G. C. Hansson , Gut 2014, 63, 281. 23426893 10.1136/gutjnl-2012-303207 PMC3740207

[5] D. Yao , W. Dai , M. Dong , C. Dai , S. Wu , EBioMedicine 2021, 74, 103751. 34902790 10.1016/j.ebiom.2021.103751 PMC8671112

[6] G. C. Hansson , Annu. Rev. Biochem. 2020, 89, 769. 32243763 10.1146/annurev-biochem-011520-105053 PMC8442341

[7] M. Zarepour , K. Bhullar , M. Montero , C. Ma , T. Huang , A. Velcich , L. Xia , B. A. Vallance , Infect. Immun. 2013, 81, 3672. 23876803 10.1128/IAI.00854-13 PMC3811786

[8] G. C. Hansson , M. E. Johansson , Gut Microbes 2010, 1, 51. 21327117 10.4161/gmic.1.1.10470 PMC3035142

[9] D. Ambort , M. E. V. Johansson , J. K. Gustafsson , H. E. Nilsson , A. Ermund , B. R. Johansson , P. J. B. Koeck , H. Hebert , G. C. Hansson , Proc. Natl. Acad. Sci. USA 2012, 109, 5645. 22451922 10.1073/pnas.1120269109 PMC3326483

[10] A. K. Singh , W. Xia , B. Riederer , M. Juric , J. Li , W. Zheng , A. Cinar , F. Xiao , O. Bachmann , P. Song , J. Praetorius , C. Aalkjaer , U. Seidler , J. Physiol. 2013, 591, 2189. 23401617 10.1113/jphysiol.2012.247874 PMC3634528

[11] P. M. Quinton , Am. J. Physiol. Cell Physiol. 2010, 299, C1222. 20926781 10.1152/ajpcell.00362.2010 PMC3006319

[12] G. M. Birchenough , M. E. Johansson , J. K. Gustafsson , J. H. Bergstrom , G. C. Hansson , Mucosal Immunol. 2015, 8, 712. 25872481 10.1038/mi.2015.32 PMC4631840

[13] G. M. Birchenough , E. E. Nystrom , M. E. Johansson , G. C. Hansson , Science 2016, 352, 1535. 27339979 10.1126/science.aaf7419 PMC5148821

[14] J. K. Gustafsson , M. E. V. Johansson , Nat. Rev. Gastroenterol. Hepatol. 2022, 19, 785. 36097076 10.1038/s41575-022-00675-x

[15] K. B. Adler , M. J. Tuvim , B. F. Dickey , Front. Endocrinol. 2013, 4, 129. 10.3389/fendo.2013.00129 PMC3776272 24065956

[16] U. Bertsch , C. Roder , H. Kalthoff , A. Trauzold , Cell Death Dis. 2014, 5, 1390. 10.1038/cddis.2014.351 PMC4454323 25165876

[17] U. Mert , A. D. Sanlioglu , Cell. Mol. Life Sci. 2017, 74, 245. 27510421 10.1007/s00018-016-2321-z PMC11107773

[18] S. von Karstedt , A. Montinaro , H. Walczak , Nat. Rev. Cancer 2017, 17, 352. 28536452 10.1038/nrc.2017.28

[19] J. Strater , P. Moller , Ann. N. Y. Acad. Sci. 2000, 915, 162. 11193573 10.1111/j.1749-6632.2000.tb05239.x

[20] J. Liu , K. Liu , Y. Wang , Z. Shi , R. Xu , Y. Zhang , J. Li , C. Liu , B. Xue , Cell Death Dis. 2024, 15, 27. 38199990 10.1038/s41419-023-06409-4 PMC10782029

[21] J. Sträter , H. Walczak , T. Pukrop , L. Von Müller , C. Hasel , M. Kornmann , T. Mertens , P. Möller , Gastroenterology 2002, 122, 659. 11874999 10.1053/gast.2002.31889

[22] J. Zhu , L. Chen , J. Shi , S. Liu , Y. Liu , D. Zheng , Immunology 2014, 141, 211. 24117005 10.1111/imm.12181 PMC3904242

[23] D. R. Halm , S. T. Halm , Am. J. Physiol. Cell Physiol. 2000, 278, C212. 10644530 10.1152/ajpcell.2000.278.1.C212

[24] G. Wang , X. Wang , H. Yu , S. Wei , N. Williams , D. L. Holmes , R. Halfmann , J. Naidoo , L. Wang , L. Li , S. Chen , P. Harran , X. Lei , X. Wang , Nat. Chem. Biol. 2013, 9, 84. 23292651 10.1038/nchembio.1153

[25] M. Espinosa , G. Noe , C. Troncoso , S. B. Ho , M. Villalon , Hum. Reprod. 2002, 17, 1964. 12151422 10.1093/humrep/17.8.1964

[26] K. Yu , R. Lujan , A. Marmorstein , S. Gabriel , H. C. Hartzell , J. Clin. Invest. 2010, 120, 1722. 20407206 10.1172/JCI41129 PMC2860923

[27] G.o Ito , R. Okamoto , T. Murano , H. Shimizu , S. Fujii , T. Nakata , T. Mizutani , S. Yui , J. Akiyama‐Morio , Y. Nemoto , E. Okada , A. Araki , K. Ohtsuka , K. Tsuchiya , T. Nakamura , M. Watanabe , PLoS One 2013, 8, 79693. 10.1371/journal.pone.0079693 PMC3818177 24223998

[28] F. Xiao , J. Li , A. K. Singh , B. Riederer , J. Wang , A. Sultan , H. Park , M. G. Lee , G. Lamprecht , B. J. Scholte , H. R. De Jonge , U. Seidler , J. Physiol. 2012, 590, 5317. 22802588 10.1113/jphysiol.2012.232124 PMC3515821

[29] E. R. McDonald III , P. C. Chui , P. F. Martelli , D. T. Dicker , W. S. El‐Deiry , J. Biol. Chem. 2001, 276, 14939. 11279061 10.1074/jbc.M100399200

[30] H. Wang , L. Xiong , P. Cramer , Proc. Natl. Acad. Sci. USA 2021, 118, 2108859118. 10.1073/pnas.2108859118 PMC8325331 34301908

[31] Y. J. Joo , S. B. Ficarro , L. M. Soares , Y. Chun , J. A. Marto , S. Buratowski , Genes Dev. 2017, 31, 2162. 29203645 10.1101/gad.306324.117 PMC5749164

[32] K. J. Wright , M. T. Marr II , R. Tjian , Proc. Natl. Acad. Sci. USA 2006, 103, 12347. 16895980 10.1073/pnas.0605499103 PMC1567882

[33] F. Salem , N. Kindt , J. R. Marchesi , P. Netter , A. Lopez , T. Kokten , S. Danese , J.‐Y. Jouzeau , L. Peyrin‐Biroulet , D. Moulin , United Eur. Gastroenterol. J. 2019, 7, 1008. 10.1177/2050640619867555 PMC6794689 31662859

[34] Y.‐W. Chao , Y.‐T. Tung , S.‐C. Yang , H. Shirakawa , L.‐H. Su , P.‐Y. Loe , W.‐C. Chiu , Nutrients 2024, 16, 980. 39275295 10.3390/nu16172980 PMC11397027

[35] P. D. Cani , C. Depommier , M. Derrien , A. Everard , W. M. de Vos , Nat. Rev. Gastroenterol. Hepatol. 2022, 19, 625. 35641786 10.1038/s41575-022-00631-9

[36] Q. Zhao , C. L. Maynard , Gut Microbes 2022, 14, 2041342. 35239459 10.1080/19490976.2022.2041342 PMC8903774

[37] P. Paone , P. D. Cani , Gut 2020, 69, 2232. 32917747 10.1136/gutjnl-2020-322260 PMC7677487

[38] T. Pelaseyed , J. H. Bergström , J. K. Gustafsson , A. Ermund , G. M. H. Birchenough , A. Schütte , S. van der Post , F. Svensson , A. M. Rodríguez‐Piñeiro , E. E. L. Nyström , C. Wising , M. E. V. Johansson , G. C. Hansson , Immunol. Rev. 2014, 260, 8. 24942678 10.1111/imr.12182 PMC4281373

[39] M. E. Johansson , M. Phillipson , J. Petersson , A. Velcich , L. Holm , G. C. Hansson , Proc. Natl. Acad. Sci. USA 2008, 105, 15064. 18806221 10.1073/pnas.0803124105 PMC2567493

[40] P. L. Ljungholm , A. Ermund , M. M. Soderlund Garsveden , V. L. Pettersson , J. K. Gustafsson , Pflugers Arch. 2024, 476, 1209. 38829391 10.1007/s00424-024-02975-4 PMC11271379

[41] M. A. McGuckin , S. K. Linden , P. Sutton , T. H. Florin , Nat. Rev. Microbiol. 2011, 9, 265. 21407243 10.1038/nrmicro2538

[42] R. C. De Lisle , J. Clin. Invest. 2009, 119, 2535. 19726878 10.1172/JCI40598 PMC2735941

[43] M. A. Garcia , N. Yang , P. M. Quinton , J. Clin. Invest. 2009, 119, 2613. 19726884 10.1172/JCI38662 PMC2735925

[44] T. E. Phillips , T. H. Phillips , M. R. Neutra , Am. J. Physiol. 1984, 247, G674. 6391203 10.1152/ajpgi.1984.247.6.G674

[45] T. E. Phillips , Am. J. Physiol. 1992, 262, G327. 1539664 10.1152/ajpgi.1992.262.2.G327

[46] G. Cantero‐Recasens , C. M. Butnaru , M. A. Valverde , J. R. Naranjo , N. Brouwers , V. Malhotra , Elife 2018, 7, 39729. 10.7554/eLife.39729 PMC6167051 30272559

[47] M. R. Sprick , M. A. Weigand , E. Rieser , C. T. Rauch , P. Juo , J. Blenis , P. H. Krammer , H. Walczak , Immunity 2000, 12, 599. 10894160 10.1016/s1074-7613(00)80211-3

[48] L. A. Grisanti , Front. Physiol. 2023, 14, 1256852. 37621762 10.3389/fphys.2023.1256852 PMC10445540

[49] A. Guerrache , O. Micheau , Cells 2024, 13, 521. 38534365 10.3390/cells13060521 PMC10968836

[50] V. Haselmann , A. Kurz , U. Bertsch , S. Hübner , M. Olempska‐Müller , J. Fritsch , R. Häsler , A. Pickl , H. Fritsche , F. Annewanter , C. Engler , B. Fleig , A. Bernt , C. Röder , H. Schmidt , C. Gelhaus , C. Hauser , J.‐H. Egberts , C. Heneweer , A. M. Rohde , C. Böger , U. Knippschild , C. Röcken , D. Adam , H. Walczak , S. Schütze , O. Janssen , F. G. Wulczyn , H. Wajant , H. Kalthoff , et al., Gastroenterology 2014, 146, 278. 24120475 10.1053/j.gastro.2013.10.009

[51] U. Mert , A. Adawy , E. Scharff , P. Teichmann , A. Willms , V. Haselmann , C. Colmorgen , J. Lemke , S. von Karstedt , J. Fritsch , A. Trauzold , Cancers 2019, 11, 1167. 31416165 10.3390/cancers11081167 PMC6721811

[52] E. C. Martens , M. Neumann , M. S. Desai , Nat. Rev. Microbiol. 2018, 16, 457. 29904082 10.1038/s41579-018-0036-x

[53] M. S. Desai , A. M. Seekatz , N. M. Koropatkin , N. Kamada , C. A. Hickey , M. Wolter , N. A. Pudlo , S. Kitamoto , N. Terrapon , A. Muller , V. B. Young , B. Henrissat , P. Wilmes , T. S. Stappenbeck , G. Núñez , E. C. Martens , Cell 2016, 167, 1339. 27863247 10.1016/j.cell.2016.10.043 PMC5131798

[54] Y. Liu , K. Fu , E. M. Wier , Y. Lei , A. Hodgson , D. Xu , X. Xia , D. Zheng , H. Ding , C. L. Sears , J. Yang , F. Wan , Cancer Discov. 2022, 12, 236. 34479870 10.1158/2159-8290.CD-21-0912 PMC8758537

[55] I. T. Chyuan , H. F. Tsai , C. S. Wu , P. N. Hsu , Mucosal Immunol. 2019, 12, 980. 31076664 10.1038/s41385-019-0168-y PMC7746525

[56] D. P. Lin , Y. L. Jin , D. Y. Hu , S. J. Ying , Y. Jiang , Am. J. Med. Sci. 2021, 362, 188. 33932348 10.1016/j.amjms.2021.04.011

[57] L. J. Cai , M. F. Wang , X. L. Wang , H. F. Zhu , X. Z. Chen , J. Biol. Regul. Homeost. Agents 2020, 34, 525. 32425017 10.23812/19-373-A

[58] Y. Yu , W. Yang , A. J. Bilotta , Y.u Yu , X. Zhao , Z. Zhou , S. Yao , J. Xu , J. Zhou , S. M. Dann , Y. Li , Y. Cong , FASEB J. 2020, 34, 15417. 32969062 10.1096/fj.202001524R PMC7606834

[59] N. Kihara , S. G. de la Fuente , K. Fujino , T. Takahashi , T. N. Pappas , C. R. Mantyh , Gut 2003, 52, 713. 12692058 10.1136/gut.52.5.713 PMC1773638

[60] S. S. Spicer , J. Histochem. Cytochem. 1965, 13, 211. 14327695 10.1177/13.3.211

[61] M. Nébot‐Vivinus , World J. Gastroenterol. 2014, 20, 6832. 24944474 10.3748/wjg.v20.i22.6832 PMC4051923

[62] T. Sato , D. E. Stange , M. Ferrante , R. G. J. Vries , J. H. van Es , S. van den Brink , W. J. van Houdt , A. Pronk , J. van Gorp , P. D. Siersema , H. Clevers , Gastroenterology 2011, 141, 1762. 21889923 10.1053/j.gastro.2011.07.050

[63] J. Zhang , Q. Yu , D. Jiang , K. Yu , W. Yu , Z. Chi , S. Chen , M. Li , D. Yang , Z. Wang , T. Xu , X. Guo , K. Zhang , H. Fang , Q. Ye , Y. He , X. Zhang , D.i Wang , Sci. Immunol. 2022, 7, abk2092. 10.1126/sciimmunol.abk2092 35119941

[64] Y. Zhang , G. Qi , X. Qu , B. Wang , K. Ma , Y. Jin , Langmuir 2022, 38, 584. 34971310 10.1021/acs.langmuir.1c03256

[65] K. Shimizu , I. Seiki , Y. Goto , T. Murata , Antibiotics 2021, 10, 180. 33670214 10.3390/antibiotics10020180 PMC7916911

