# Supplementary material for: DR5 Governs Compound Exocytosis in Colonic Goblet Cells via TATA‐Box Binding Protein‐Dependent Bestrophin‐2 Transcriptional Regulation
Source: Adv Sci (Weinh). 2025 Nov 11;13(6):e16789. doi: 10.1002/advs.202516789 (PMC12866826; doi:10.1002/advs.202516789)
Supplement: Supplementary file 1 — Supporting Information [file ADVS-13-e16789-s001.docx]

Supporting Information

Title

DR5 governs compound exocytosis in colonic goblet cells via TATA-box binding protein-dependent Bestrophin-2 transcriptional regulation

**Author(s), and Corresponding Author(s)***

Ying Wang^1^, Xinyun Li^1 2^, Yong Wang^1^, Chuhe Yang^1^, Xiaopei Gao^1^, Ke Zhu^1^, Yihang Ren^1^, Jingxin Li^1^, Chuanyong Liu^1^ and Bing Xue^1*^


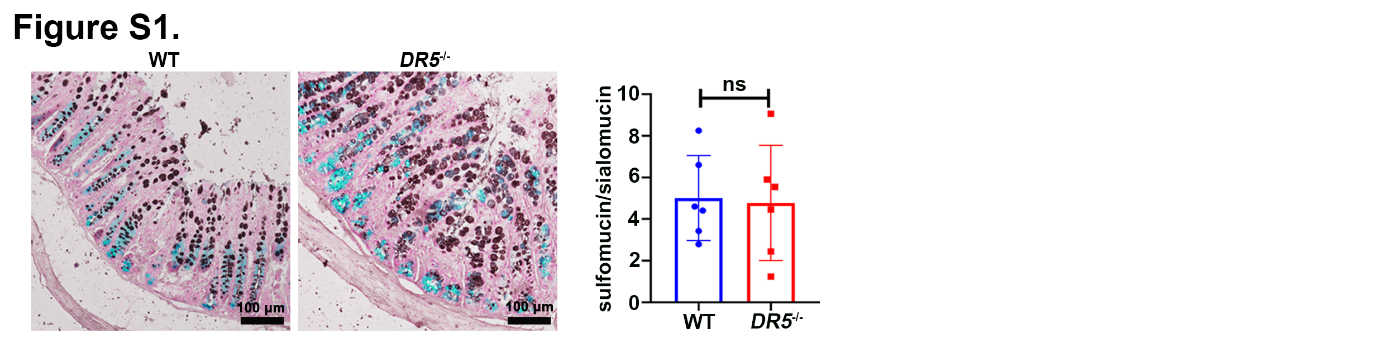


**Figure S1. *DR5* Knockout does not alter colonic mucin composition.** Representative images of high iron diamine/Alcian blue (HID/AB)-stained colon sections from WT and *DR5*^-/-^ mice and quantification of the sulfomucin-to-sialomucin ratio in colonic goblet cells. The ratio was determined by analyzing five randomly selected 200× magnification fields of view per mouse (*n* = 6). Data are expressed as mean ± SD. Data was analyzed by unpaired *t*-test. Ns indicates not significant.


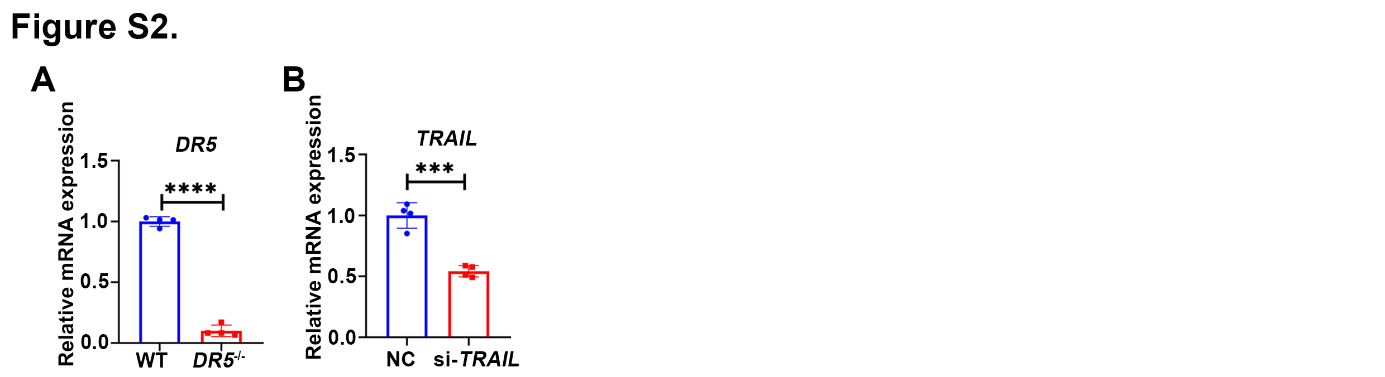


**Figure S2. Validation of target gene knockout/knockdown in colonic organoids.** A) qRT-PCR analysis of *DR5* knockout efficiency in WT and *DR5*^-/-^ organoids (*n* = 4). B) qRT-PCR analysis of *TRAIL* knockdown efficiency in colonic organoids. Colonic organoids cultured for 5 days were transfected with si-*TRAIL* or NC (*n* = 4). Data are expressed as mean ± SD. All data were analyzed by unpaired *t*-test. ^***^*P* < 0.001, ^****^*P* < 0.0001.


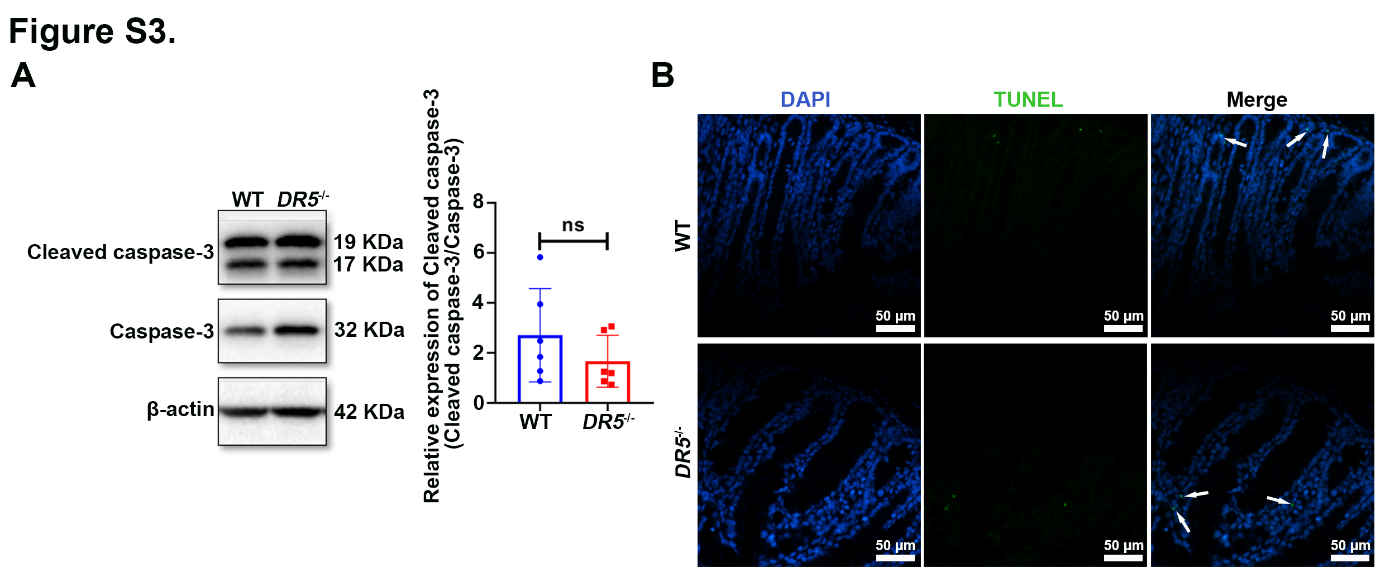


**Figure S3. *DR5* knockout does not affect colonic cell apoptosis.** A) Representative western blot and quantification of Cleaved caspase-3 protein levels in the colon of WT and *DR5*^-/-^ mice (*n* = 6). B) Immunofluorescence images of terminal deoxynucleotidyl transferase-mediated dUTP nick-end labelling (TUNEL) staining in colon sections from WT and *DR5*^-/-^ mice. Data are expressed as mean ± SD. Data was analyzed by unpaired *t*-test. Ns indicates not significant.


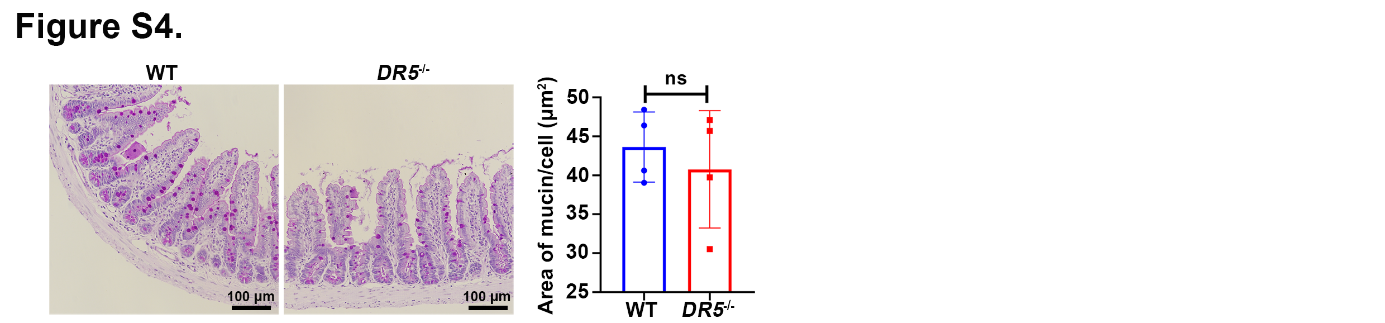


**Figure S4. Absence of mucin over-accumulation in ileal goblet cells of *DR5*^-/-^ mice.** Representative periodic acid–Schiff (PAS)-stained sections of ileum and quantification of the mucin area per goblet cell in >10 randomly selected crypts per section (*n* = 4). Data are expressed as mean ± SD. Data was analyzed by unpaired *t*-test. Ns indicates not significant.


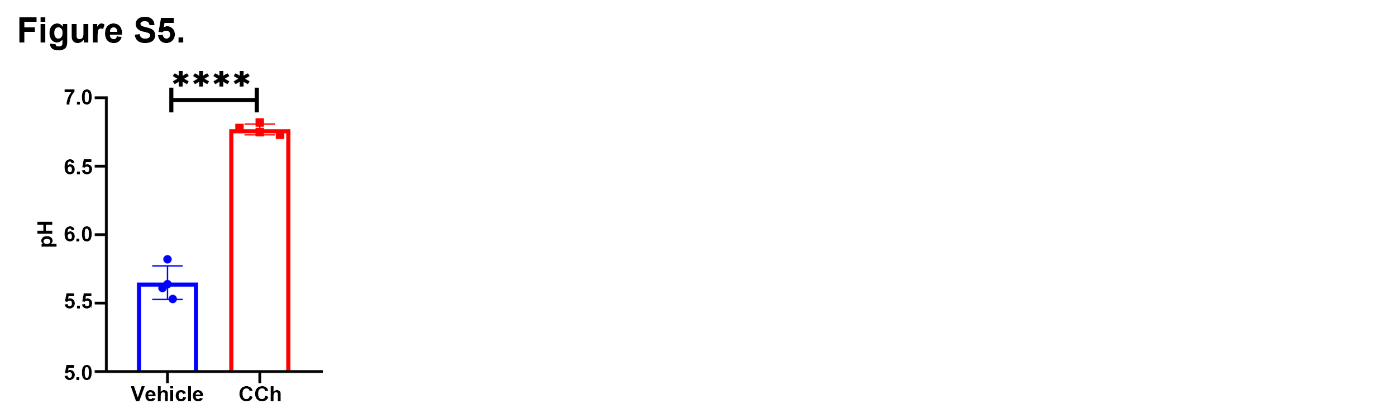


**Figure S5. CCh stimulation increases the supernatant pH in ex vivo colonic explants.** Supernatant pH was measured in ex vivo colonic explants from WT mice following 10 min of exposure to vehicle or 1 mm CCh, reflecting stimulated HCO₃^-^ secretion (*n* = 4). Data are expressed as mean ± SD. Data was analyzed by unpaired *t*-test. ^***^*P* < 0.001.


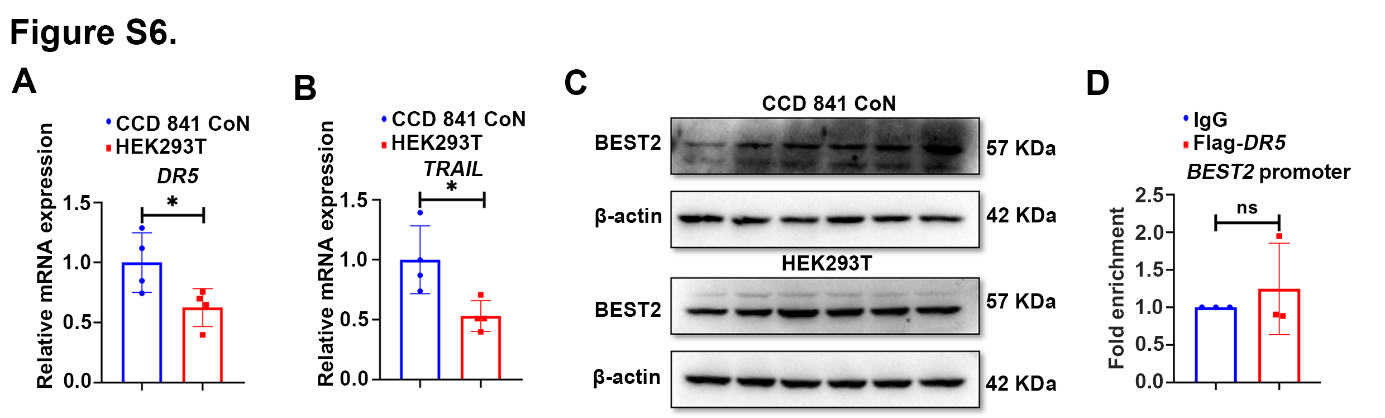


**Figure S6. *DR5*, *TRAIL* and BEST2 expression in CCD 841 CoN and HEK293T cells, and CHIP-qPCR analysis of DR5 binding to the *BEST2* promoter in HEK293T cells.** A) qRT-PCR analysis of *DR5* mRNA in CCD 841 CoN and HEK293T cells (*n* = 4). B) qRT-PCR analysis of *TRAIL* mRNA in CCD 841 CoN and HEK293T cells (*n* = 4). C) Western blot analysis of BEST2 expression in CCD 841 CoN and HEK293T cells (*n* = 6). D) HEK293T cells were transfected with Flag-*DR5* plasmid and then stimulated with Bioymifi (100 nm) for 72 h before ChIP using anti-Flag antibody (*n* =3). Data are expressed as mean ± SD. All data were analyzed by unpaired *t*-test. Ns indicates not significant. ^*^*P* < 0.05.


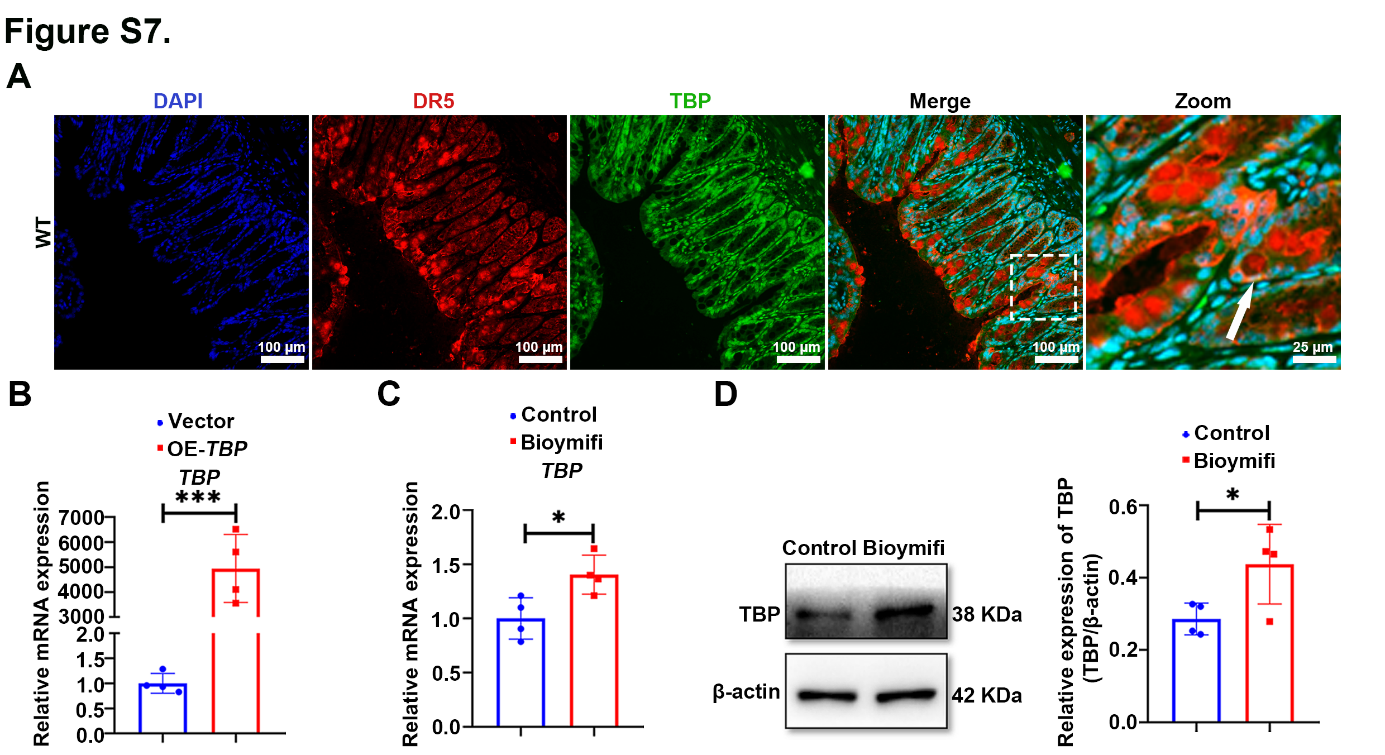


**Figure S7. Supporting evidence for TBP in DR5-dependent regulation of Best2 transcription.** A) Immunofluorescence staining of DR5 and TBP in colon sections. White arrow indicates goblet cell showing nuclear co-localization of DR5 and TBP. B) qRT-PCR analysis of *TBP* mRNA expression in HEK293T cells transfected with *TBP* plasmid or vector (*n* = 4). C) qRT-PCR analysis of *TBP* mRNA in CCD 841 CoN cells stimulated with Bioymifi (100 nm, 48 h) (*n* = 4). D) Western blot analysis of TBP expression in CCD 841 CoN cells stimulated with Bioymifi (100 nm, 48 h) (*n* = 4). Data are expressed as mean ± SD. All data were analyzed by unpaired *t*-test. ^*^*P* < 0.05, ^***^*P* < 0.001.


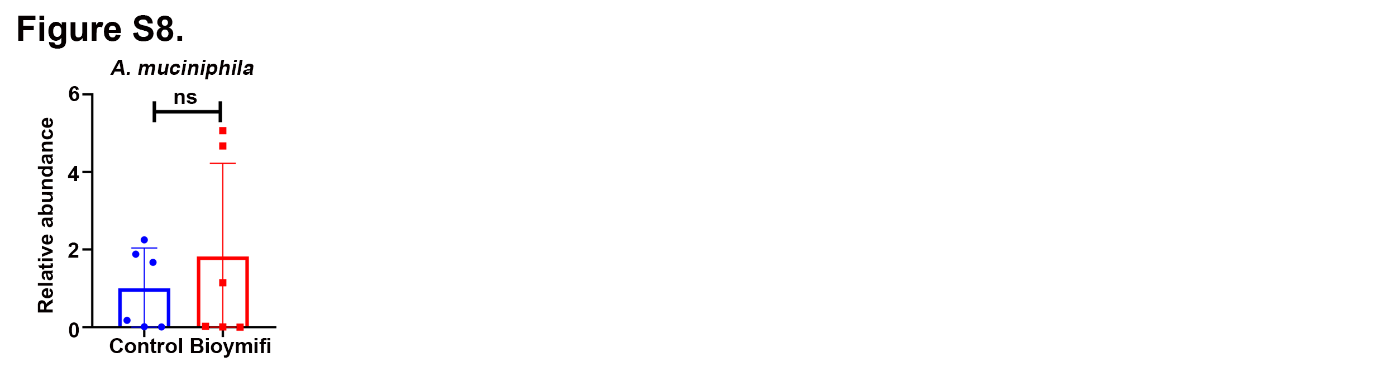
**Figure S8. Fecal abundance of *Akkermansia muciniphila* is unaltered by Bioymifi treatment in vivo.** Mice received daily intracolonic infusion of Bioymifi (100 nm in 200 μL) or vehicle for 7 days. Fecal abundance of *Akkermansia muciniphila* was quantified by qPCR (*n* = 6). Data are expressed as mean ± SD. Data was analyzed by unpaired t-*t*est. Ns indicates not significant.


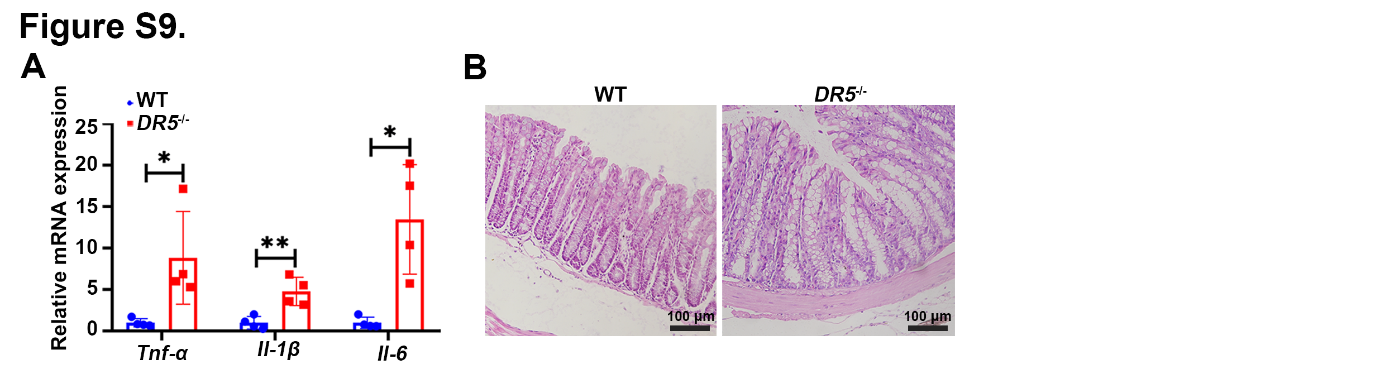


**Figure S9. *DR5* knockout upregulates pro-inflammatory cytokine expression but does not alter tissue histology.** A) qRT-PCR analysis of the inflammatory cytokine mRNA expression in the colon of WT and *DR5*^-/-^ mice (*n* = 4). B) Images of Hematoxylin and eosin (H&E) colon sections in WT and *DR5*^-/-^ mice. Data are expressed as mean ± SD. Data were analyzed by unpaired *t*-test. ^*^*P* < 0.05, ^**^*P* < 0.01.

**Table S1. Prediction of the transcription factors for *BEST2***

| **GTRD database** | **JASPAR database** | **HumanTFDB**  **database** | **Common transcription factors** |
| --- | --- | --- | --- |
| IKZF3 | SRF | GTF3C2 | SRF |
| ELK4 | Ar | GLYR1 | E2F1 |
| SAP130 | E2F1 | KLF1 | NFKB1 |
| SMARCB1 | NFKB1 | RCOR1 | NR2F1 |
| CEBPB | NR2F1 | FOXA2 | TBP |
| SP7 | Nr2e3 | POLR3A | NFE2L2 |
| SMAD3 | Myb | SPI1 | SPI1 |
| MSC | Klf4 | LMNB1 | PPARG |
| STAG2 | PLAG1 | ETV4 | SP1 |
| CXXC4 | TBP | ESR1 | RELA |
| PRDM11 | Egr1 | FOXG1 | ESR1 |
| PRDM10 | MZF1_5-13 | PCGF2 | TP53 |
| PRDM9 | INSM1 | MAZ | BRCA1 |
| FXR1 | NFE2L2 | SUZ12 | ZEB1 |
| RAD21 | RREB1 | TCF12 | EBF1 |
| ZEB1 | NHLH1 | CDK9 | HNF4A |
| TRIM22 | Nobox | ZNF263 | CEBPA |
| PEX2 | MZF1_1-4 | REST | ETS1 |
| SIRT1 | SPI1 | MYC | GATA3 |
| AGO2 | NF-kappaB | POU5F1 | CREB1 |
| TEAD3 | Spz1 | DDX5 | YY1 |
| ZBTB2 | ELK1 | EZH2 | GATA2 |
| PTBP1 | Mafb | IRF5 | TEAD1 |
| PBX1 | EWSR1-FLI1 | TAF1 | RUNX1 |
| RFX5 | NFIC | FLI1 | CTCF |
| HNRNPH1 | PPARG | EGR2 | PBX1 |
| NFKB1 | SP1 | TRIM28 | GABPA |
| DTL | T | KLF5 | IRF2 |
| U2AF2 | RELA | CDK7 | FOXA1 |
| RUNX2 | ESR1 | C17orf96 | STAT1 |
| RCOR2 | TP53 | CTBP2 | MAX |
| AGO1 | Pdx1 | FOXO3 | NFYA |
| LEO1 | Pax5 | KLF4 | USF1 |
| TBX21 | Foxq1 | HDAC2 | NR3C1 |
| RING1 | Pax4 | KLF9 | REST |
| KLF17 | HNF1A | SP3 |  |
| ZNF48 | BRCA1 | E2F1 |  |
| BRD4 | RORA_2 | SP1 |  |
| T-Cell | ZEB1 | TRIM24 |  |
| BHLHE40 | ESR2 | TCF4 |  |
| CREM | EBF1 | CDK8 |  |
| HDAC2 | N/A | TCF3 |  |
| EHF | HNF4A | EP300 |  |
| ZBTB7A | Zfp423 | SRF |  |
| RBPJ | znf143 | SP4 |  |
| OR2M7 | TFAP2A | POLR2A |  |
| HOXB8 | SPIB | EGR1 |  |
| UBTF | Foxd3 | ZNF250 |  |
| EZH2 | Prrx2 | KLF15 |  |
| SOX13 | SOX10 | ZNF92 |  |
| TP53BP1 | Gfi | BRD4 |  |
| BRCA1 | Zfx | YY1 |  |
| TFAP2C | Pou5f1 | FOXK1 |  |
| THRB | CEBPA | AR |  |
| CHD4 | ETS1 | USF1 |  |
| NFKBIZ | FOXC1 | SMARCA4 |  |
| ZBTB26 | GATA3 | ZNF384 |  |
| GABPA | CREB1 | GTF2I |  |
| GABPB1 | NR4A2 | HIF1A |  |
| FOXA2 | FOXD1 | ZBTB33 |  |
| NR3C1 | YY1 | NR2F2 |  |
| KDM5B | GATA2 | ZNF148 |  |
| TEAD1 | TEAD1 | HOXA6 |  |
| BRD9 | SRY | CBFB |  |
| RCOR1 | RUNX1 | TBP |  |
| ZNF574 | Esrrb | TFAP4 |  |
| LARP7 | Pax2 | BCL3 |  |
| IRF2 | Sox2 | RREB1 |  |
| TCF4 | SOX9 | SP2 |  |
| GATAD1 | Hltf | TBL1XR1 |  |
| SUZ12 | NKX3-1 | E2F5 |  |
| ZC3H8 | FEV | ETS1 |  |
| ZBTB33 | ELK4 | BMI1 |  |
| ARNTL | NFIL3 | CHD1 |  |
| NSD2 | FOXI1 | SMAD1 |  |
| EP300 | CTCF | SRC |  |
| RBFOX2 | FOXL1 | EWSR1 |  |
| ETV2 | Myc | E2F7 |  |
| NFKB2 | Myf | GTF2B |  |
| SOX8 | ZNF354C | ZBTB7B |  |
| SETDB1 | REL | WT1 |  |
| SFPQ | PBX1 | PHF8 |  |
| BRD2 | HOXA5 | SUMO2 |  |
| E2F8 | GABPA | E2F4 |  |
| CRTC2 | Mycn | BDP1 |  |
| ZNF664 | Stat3 | JUND |  |
| CTCFL | MEF2A | BTAF1 |  |
| SMARCA4 | RORA_1 | MYB |  |
| BMI1 | IRF2 | HSF1 |  |
| ZNF263 | Nkx2-5 | ORC1 |  |
| SIN3A | Sox17 | VEZF1 |  |
| CBFA2T3 | HLF | NR3C1 |  |
| PTEN | Pax6 | GRHL3 |  |
| RBBP5 | HNF1B | RNF2 |  |
| BRD3 | Tcfcp2l1 | ZNF423 |  |
| RXRB | FOXA1 | FOXP1 |  |
| KLF4 | STAT1 | BRD3 |  |
| YY1 | FOXF2 | CREB1 |  |
| CDK7 | MAX | KDM1A |  |
| ZNF639 | FOXO3 | ZNF143 |  |
| GTF3C5 | ARID3A | TAF3 |  |
| ELK3 | MIZF | BCL11A |  |
| JUN | Arnt | TP73 |  |
| CENPA | NFYA | SIN3A |  |
| PPARG | USF1 | TFAP2A |  |
| SMARCC1 | NR3C1 | NOTCH1 |  |
| ZNF366 | NFATC2 | ERG |  |
| ZNF770 | ELF5 | TTF1 |  |
| KMT2B | IRF1 | PLAG1 |  |
| XRCC5 | REST | EHF |  |
| ZNF395 | Foxa2 | ARNT2 |  |
| ZNF614 | Evi1 | RELA |  |
| GATA6 | Sox5 | MXI1 |  |
| MEIS3P1 | AP1 | IRF3 |  |
| GTF2F1 | En1 | ZEB1 |  |
| MITF | Lhx3 | NR2C2 |  |
| NEUROG2 | Gata1 | RUNX2 |  |
| UPF1 | Nkx3-2 | BRD7 |  |
| ZKSCAN8 |  | ZNF280D |  |
| ZMYM3 |  | ELK1 |  |
| DEK |  | ARRB1 |  |
| SRSF7 |  | IRF1 |  |
| BCL11A |  | KMT2A |  |
| MYRF |  | POU2F1 |  |
| SP1 |  | MYOD1 |  |
| CTBP1 |  | TP53 |  |
| REST |  | GATA1 |  |
| NCOA1 |  | ASCL1 |  |
| TAF7 |  | BHLHE40 |  |
| ZBTB42 |  | ZFX |  |
| POU2AF1 |  | TFAP2C |  |
| IRF4 |  | MAFB |  |
| NCOR1 |  | NFYB |  |
| RARG |  | EPAS1 |  |
| SP4 |  | MBD3 |  |
| NOTCH1 |  | NR2F1 |  |
| SMAD2 |  | COUP-TF:HNF4 |  |
| SUMO2 |  | NCOR1 |  |
| NFIL3 |  | EZH1 |  |
| SMAD1 |  | KDM5B |  |
| FUS |  | MNX1 |  |
| STAG1 |  | GATA4 |  |
| HIRA |  | NANOG |  |
| PHF8 |  | SOX2 |  |
| HOXC9 |  | UBTF |  |
| SKI |  | TAL1 |  |
| ZNF121 |  | ARNT |  |
| CDKN1B |  | E2F3 |  |
| CHD7 |  | HAND1 |  |
| TEAD4 |  | KLF13 |  |
| USP7 |  | NHLH1 |  |
| TARDBP |  | GATA3 |  |
| PBX4 |  | TOP1 |  |
| AHR |  | STAT4 |  |
| NRF1 |  | BRCA1 |  |
| NR2F6 |  | RXRA |  |
| NR2F1 |  | BCL6 |  |
| SREBF1 |  | NFKB1 |  |
| FOXH1 |  | NFATC1 |  |
| ZNF554 |  | LRF |  |
| HIF1A |  | FOXA1 |  |
| HNRNPK |  | USF2 |  |
| RBM39 |  | VDR |  |
| KDM6B |  | TTF2 |  |
| SCRT1 |  | PPARG |  |
| ZNF35 |  | PGR |  |
| ZNF652 |  | WHSC1 |  |
| GRHL3 |  | ELF1 |  |
| MBL2 |  | NFE2 |  |
| SUPT6H |  | STAT2 |  |
| FLI1 |  | HDAC6 |  |
| RBM14 |  | STAT3 |  |
| CBX3 |  | CTCF |  |
| EOMES |  | IKZF1 |  |
| PCBP2 |  | ELK3 |  |
| ZFP64 |  | RB1 |  |
| PCBP1 |  | PBX1 |  |
| RBBP4 |  | FOSL1 |  |
| ESRRA |  | PPARD/PPARG |  |
| VDR |  | AP4 |  |
| SLC30A9 |  | NOBOX |  |
| ELF3 |  | SREBF2 |  |
| PHF6 |  | STAT1 |  |
| EP400 |  | OTX2 |  |
| KDM5A |  | SMAD3 |  |
| OLIG2 |  | EOMES |  |
| ZSCAN5A |  | REPIN1 |  |
| MTOR |  | PAX5 |  |
| KDM3A |  | SETDB1 |  |
| THAP11 |  | CREBBP |  |
| HMGXB4 |  | RAC3 |  |
| ARID4B |  | ZBTB7A |  |
| RUNX3 |  | EGR3 |  |
| EED |  | T |  |
| YAP1 |  | SIX5 |  |
| GRHL2 |  | RFX2 |  |
| ZNF341 |  | ESRRA |  |
| EBF1 |  | MED1 |  |
| EGR3 |  | SUMO2/SUMO3 |  |
| BAP1 |  | DBP |  |
| BCHE |  | SOAT1 |  |
| CBX1 |  | FOXP3 |  |
| RXRA |  | CEBPA |  |
| GATAD2B |  | SREBF1 |  |
| RUNX1 |  | INSM1 |  |
| HNF4A |  | NFYA |  |
| CREBBP |  | HES1 |  |
| HDGFL2 |  | ZBTB17 |  |
| FIP1L1 |  | NEUROD1 |  |
| MBD3 |  | P50:P50 |  |
| CTBP2 |  | SOX4 |  |
| ZNF280A |  | SNAI2 |  |
| HDAC1 |  | SRY |  |
| ELF1 |  | FOXO1 |  |
| CBX6 |  | TBL1X |  |
| HOMEZ |  | ZC3H8 |  |
| SRF |  | PAX6 |  |
| RELA |  | RARG |  |
| DPF2 |  | SMAD2/SMAD3 |  |
| RFXANK |  | PBX3 |  |
| MECOM |  | STAT5A |  |
| LMO1 |  | HCFC1 |  |
| NEUROD1 |  | MAX |  |
| SRCAP |  | AIRE |  |
| SSU72 |  | MZF1 |  |
| ETS1 |  | SOX17 |  |
| LYL1 |  | NRF1 |  |
| TET2 |  | LMO2 |  |
| EBF3 |  | BRD2 |  |
| ZNF644 |  | PML |  |
| GATA4 |  | ELL2 |  |
| BICRA |  | NR5A2 |  |
| SP140 |  | HOXA7 |  |
| MLXIP |  | HEY1 |  |
| NKX2-1 |  | THAP1 |  |
| RAG2 |  | CACD |  |
| FOXP1 |  | MYH11 |  |
| CDK2 |  | HIC1 |  |
| TCF7L1 |  | PPARA |  |
| NFE2 |  | ESR2 |  |
| SS18 |  | NR2C1 |  |
| KLF1 |  | ZFP42 |  |
| TAF1 |  | SMAD2 |  |
| SMC1A |  | FOXH1 |  |
| ZFP1 |  | TEAD4 |  |
| MBD2 |  | TFAP2B |  |
| SUPT5H |  | PDX1 |  |
| CREB1 |  | ZNF350 |  |
| CLOCK |  | PRKDC |  |
| DNMT3B |  | RARA |  |
| ZNF511 |  | GATA2 |  |
| OSR2 |  | HSF |  |
| OGG1 |  | ELSPBP1 |  |
| MAFF |  | HDAC3 |  |
| MIER3 |  | AP2 |  |
| NCAPH2 |  | RUNX3 |  |
| NELFE |  | GABPA |  |
| LEF1 |  | GMEB2 |  |
| NR2C2 |  | CBX3 |  |
| ESR1 |  | ETS2 |  |
| PGR |  | IRF4 |  |
| ZSCAN9 |  | PURA |  |
| RB1 |  | PIAS1 |  |
| KDM6A |  | COL11A2 |  |
| JUND |  | SMAD4 |  |
| ATF7 |  | RBL2 |  |
| TAL1 |  | RUNX1 |  |
| ZNF654 |  | BRF1 |  |
| DACH1 |  | SPIB |  |
| SFMBT1 |  | MYOG |  |
| GTF3C2 |  | TBX2 |  |
| SCRT2 |  | JUN |  |
| MYOG |  | HEY2 |  |
| MYOD1 |  | RP58 |  |
| CAT |  | NF1 |  |
| SRSF3 |  | GLI3 |  |
| ELL2 |  | ZNF219 |  |
| RUNX1T1 |  | E2F6 |  |
| MBD4 |  | MITF |  |
| WT1 |  | PPARG:RXRA |  |
| CUL4A |  | RBPJ |  |
| RORC |  | ZNF711 |  |
| KAT7 |  | ZNF76 |  |
| SP2 |  | SOX18 |  |
| MAX |  | FOXF1 |  |
| TBL1XR1 |  | ZBED6 |  |
| ZMYND8 |  | TLX1:NFIC |  |
| CHD2 |  | CTF1 |  |
| SREBF2 |  | RAD21 |  |
| FOXO1 |  | SALL4 |  |
| STAT5B |  | NR1I3 |  |
| KMT2C |  | KLF3 |  |
| MEN1 |  | STAG1 |  |
| KMT2A |  | EGR4 |  |
| ARID2 |  | REX1 |  |
| BCL3 |  | BPTF |  |
| RNF2 |  | NFE2L2 |  |
| INTS11 |  | GFI1B |  |
| TP53 |  | TWIST1 |  |
| TRPS1 |  | RFX3 |  |
| SPI1 |  | SNAI1 |  |
| LDB1 |  | SMC3 |  |
| NFE2L2 |  | NR0B1 |  |
| BCL11B |  | KLF8 |  |
| ZHX2 |  | ETV7 |  |
| HNRNPC |  | THAP11 |  |
| ETV1 |  | CDX1 |  |
| PRMT1 |  | CDX2 |  |
| PAX2 |  | NKX2-1 |  |
| MYC |  | PLAGL1 |  |
| PRPF4 |  | FOXP2 |  |
| FOS |  | SMAD2:SMAD3:SMAD4 |  |
| HNRNPLL |  | STAT6 |  |
| ZNF467 |  | SUMO1 |  |
| ASCL1 |  | HOXC5 |  |
| TOP1 |  | TCF21 |  |
| POU5F1 |  | LTF |  |
| RBM25 |  | STAT5B |  |
| CDX2 |  | E2F4:DP1 |  |
| E2F4 |  | RXRG |  |
| ZFP36 |  | ASCL2 |  |
| ZNF143 |  | CTCFL |  |
| KLF6 |  | HNF4G |  |
| ZNF680 |  | GLI1 |  |
| MED12 |  | PATZ1 |  |
| EGR2 |  | FOS |  |
| ZSCAN22 |  | SMC1A |  |
| ZBTB48 |  | E2F2 |  |
| NR2F2 |  | PPARGC1A |  |
| HOXB6 |  | HSF2 |  |
| AFF1 |  | PRDM1 |  |
| ILF3 |  | PPAR:HNF4:COUP:RAR |  |
| AFF4 |  | PTEN |  |
| GFI1 |  | ATF3 |  |
| CDK8 |  | MLXIPL |  |
| ZBTB25 |  | BARX2 |  |
| STAT3 |  | SP1:SP3 |  |
| PRDM14 |  | ERMAP |  |
| TAF15 |  | HNF4A |  |
| CNOT3 |  | FOXJ3 |  |
| ATF2 |  | TBX3 |  |
| NBN |  | TGIF1 |  |
| SIN3B |  | GLIS3 |  |
| ATF3 |  | HAND2 |  |
| CDK9 |  | GFI1 |  |
| KDM4A |  | ZNF274 |  |
| RBM22 |  | RUNX1T1 |  |
| BRD7 |  | RFX5 |  |
| MECP2 |  | HDAC1 |  |
| DCP1A |  | HLTF |  |
| TP63 |  | NCOR2 |  |
| ARNT |  | FOXM1 |  |
| SIRT6 |  | MYF6 |  |
| HCFC1 |  | GLI2 |  |
| ZC3H11A |  | CREM |  |
| HIF3A |  | POU1F1 |  |
| NUP98 |  | HOXB4 |  |
| DMC1 |  | LRH1 |  |
| PHF5A |  | MECP2 |  |
| TCF3 |  | ETV1 |  |
| SMAD4 |  | ZKSCAN1 |  |
| SAFB |  | MTF1 |  |
| TCF21 |  | RFX1 |  |
| MYB |  | NFIC |  |
| MYBL2 |  | NFAT5 |  |
| CDK12 |  | MTA3 |  |
| BATF |  | ZBTB18 |  |
| PCF11 |  | MIF1 |  |
| KDM2B |  | LEF1 |  |
| TWIST1 |  | DEAF1 |  |
| ZNF148 |  | NFE4 |  |
| SCML2 |  | TLX1 |  |
| ERG |  | POU2F2 |  |
| MXD3 |  | XBP1 |  |
| OTX2 |  | TBX5 |  |
| CPSF3 |  | CBX4 |  |
| ZNF384 |  | BACH1 |  |
| RBAK |  | EBF1 |  |
| GATA2 |  | FOXO4 |  |
| ZBTB20 |  | ELF2 |  |
| TRIM28 |  | CBX1 |  |
| VEZF1 |  | ETV5 |  |
| H2AFZ |  | HESX1 |  |
| NCOR2 |  | ZNF589 |  |
| SP5 |  | PTF1A |  |
| PAX5 |  | THRA |  |
| TLE3 |  | MEIS1 |  |
| GATA3 |  | THRB |  |
| MAZ |  | E2F1:DP1 |  |
| TRIM24 |  | E2F1:DP2 |  |
| HDGFL3 |  | E2F4:DP2 |  |
| E2F6 |  | PAX3 |  |
| AR |  | FUBP1 |  |
| RARA |  | MRE11A |  |
| ZBTB8A |  | ESRRG |  |
| RELB |  | FOXD3 |  |
| GLIS1 |  | KDM5A |  |
| ME3 |  | HOXC6 |  |
| HEY1 |  | KAT2B |  |
| GATA1 |  | VDR:CAR:PXR |  |
| ZNF792 |  | E2F8 |  |
| TCF7L2 |  | KLF6 |  |
| NONO |  | POU4F2 |  |
| SAP30 |  | CART1 |  |
| MAF |  | CENPA |  |
| TFAP4 |  | PRDM14 |  |
| CTCF |  | MAFA |  |
| FANCD2 |  | ARID3A |  |
| ZIC5 |  | LYL1 |  |
| CEBPA |  | CEBPB |  |
| FOXM1 |  | NFKB2 |  |
| SNAI2 |  | ICE1 |  |
| HNRNPL |  | EGLN2 |  |
| MCM7 |  | CAMTA2 |  |
| E2F1 |  | CIITA |  |
| ZKSCAN1 |  | ZIC3 |  |
| STAT1 |  | MYBL2 |  |
| JUNB |  | KLF11 |  |
| SUMO1 |  | HOXC4 |  |
| STAT5A |  | FOXA3 |  |
| CBX5 |  | TEAD2 |  |
| KLF9 |  | PRAME |  |
| HEXIM1 |  | POU3F1 |  |
| HMG20B |  | ICE2 |  |
| U2AF1 |  | JMJD6 |  |
| KLF5 |  | MEF2A |  |
| FOXA3 |  | HNF1A |  |
| FOXA1 |  | FOSL2 |  |
| KAT8 |  | KAT8 |  |
| TCF12 |  | P50:RELA-P65 |  |
| SSRP1 |  | NKX2-5 |  |
| APP |  | TEAD1 |  |
| ORC2 |  | TEF |  |
| CCAR2 |  | FGF9 |  |
| ZFX |  | TAL1:TCF3 |  |
| HDAC3 |  | TFDP1 |  |
| KLF16 |  | HOXA3 |  |
| IKZF1 |  | IRF8 |  |
| MTA2 |  | HNF1B |  |
| ZNF22 |  | ATF2 |  |
| ZBTB14 |  | CLOCK |  |
| ZNF16 |  | TP63 |  |
| BDP1 |  | ATF6 |  |
| MYCN |  | ZIC1 |  |
| USF2 |  | BCOR |  |
| JMJD6 |  | ZBTB4 |  |
| BCL6 |  | PR:GR |  |
| CEBPG |  | PALB2 |  |
| USF1 |  | HNF4:COUP |  |
| ERCC6 |  | NR1I2 |  |
| MED26 |  | OTX1 |  |
| ZBTB40 |  | HIRA |  |
| SMC3 |  | RBCK1 |  |
| NFYA |  | ARNTL |  |
| ZIC2 |  | MAFK |  |
| PMEPA1 |  | DMRT3 |  |
| HNF4G |  | IRF2 |  |
| ETV5 |  | TCF7L2 |  |
| EGR1 |  | ETV3 |  |
| SOX6 |  | DLX3 |  |
| SOX5 |  | ZBTB10 |  |
| ZNF444 |  | DMRTA1 |  |
| KDM1A |  | ISL1 |  |
| CCNT2 |  | SOX10 |  |
| MUC22 |  | BACH2 |  |
| GATAD2A |  | HOXA9 |  |
| TBP |  | BHLHE41 |  |
| ZGPAT |  | NR4A1 |  |
| SOX4 |  | CASP8AP2 |  |
| ARID1A |  | TFAP2D |  |
| HMGN3 |  | NFATC4 |  |
|  |  | IRF9 |  |
|  |  | PITX1 |  |
|  |  | AP1 |  |
|  |  | SREBP1 |  |
|  |  | RARB |  |
|  |  | KAT5 |  |
|  |  | NRIP1 |  |
|  |  | VSX2 |  |
|  |  | PLAU |  |
|  |  | ZNF217 |  |
|  |  | STAT2:STAT1 |  |
|  |  | ZIC2 |  |
|  |  | FOXC1 |  |
|  |  | POU6F1 |  |
|  |  | LHX2 |  |
|  |  | NR2E3 |  |
|  |  | MEF2C |  |
|  |  | EED |  |
|  |  | ELF3 |  |
|  |  | STAT3:STAT3 |  |
|  |  | NIPBL |  |
|  |  | MSX2 |  |
|  |  | LIN9 |  |
|  |  | CXXC1 |  |
|  |  | NF1A |  |
|  |  | SAP30 |  |
|  |  | REL |  |
|  |  | LXR:PXR:CAR:COUP:RAR |  |
|  |  | HOXB7 |  |
|  |  | DUX4 |  |
|  |  | NFE2:MAF |  |

**Table S2. Information about antibodies used in this study**

| **Antibodies for immunohistochemistry** | **Source** | **Identifier** | **Dilution ratio** |
| --- | --- | --- | --- |
| DR5 | Abcam | ab8416 | 1/200 |
| Muc2 | Abcam | ab272692 | 1/2000 |
| **Primary antibodies for immunofluorescence** | **Source** | **Identifier** | **Dilution ratio** |
| Muc2 | Abcam | ab272692 | 1/400 |
| DR5 | Abways Technology | AB3251 | 1/100 |
| DR5 | Abcam | ab8416 | 1/100 |
| TRAIL | Absin Bioscience Inc | abs121750 | 1/200 |
| Bestrophin-2 | Sant Cruz | sc-376351 | 1/200 |
| TBP | Sant Cruz | sc-421 | 1/200 |
| **Secondary antibodies for immunofluorescence** | **Source** | **Identifier** | **Dilution ratio** |
| Rhodamine (TRITC)-conjugated Goat Anti-Rabbit IgG (H + L) | Proteintech | SA00007-2 | 1/100 |
| CoraLite488-conjugated Affinipure Goat Anti-Rabbit IgG (H + L) | Proteintech | SA00013-2 | 1/100 |
| Rhodamine (TRITC)-conjugated Goat Anti-Mouse IgG (H + L) | Proteintech | SA00007-1 | 1/100 |
| CoraLite488-conjugated Affinipure Goat Anti-Mouse IgG (H + L) | Proteintech | SA00013-1 | 1/100 |
| Multi-rAb® CoraLite® Plus 594-Goat Anti-Rabbit Recombinant Secondary Antibody (H+L) | Proteintech | RGAR004 | 1/100 |
| **Primary antibodies for western blot** | **Source** | **Identifier** | **Dilution ratio** |
| β-actin | Proteintech | 66009-1-lg | 1/5000 |
| Muc2 | Abcam | ab272692 | 1/1000 |
| DR5 | Abcam | ab8416 | 1/1000 |
| TBP | Sant Cruz | sc-421 | 1/1000 |
| Cleaved caspase-3 | Cell Signaling Technology | #9662 | 1/1000 |
| Caspase-3 | Proteintech | 19677-1-AP | 1/1000 |
| **Secondary antibodies for western blot** | **Source** | **Identifier** | **Dilution ratio** |
| HRP-conjugated Goat Anti-Rabbit IgG(H+L) | Proteintech | SA00001-2 | 1/5000 |
| HRP-conjugated Goat Anti-Mouse IgG(H+L) | Proteintech | SA00001-1 | 1/5000 |

**Table S3. Target-specific siRNAs used in this study**

| **Target Gene** | **Species** | **Sequence (5'→3')** | **Strand** |
| --- | --- | --- | --- |
| *DR5* | Human | CAGCCGUAGUCUUGAUUGUTT | Sense |
|  |  | ACAAUCAAGACUACGGCUGTT | Antisense |
| *TRAIL* | Mouse | GUGCAGUACAUCUACAAGUTT | Sense |
|  |  | ACUUGUAGAUGUACUGCACTT | Antisense |
| *Best2* | Mouse | GUCGCUGUUAAGGCGUAAATT | Sense |
|  |  | UUUACGCCUUAACAGCGACTT | Antisense |
| *TBP* | Mouse | GUAGCUAUGAGCCAGAAUUTT | Sense |
|  |  | AAUUCUGGCUCAUAGCUACTT | Antisense |

**Table S4. Sequence of primers used in mRNA expression analyses**

| **Gene name** | **Sequence (5' to 3')** |
| --- | --- |
| *β-actin* (Mouse) | Forward: GGCTGTATTCCCCTCCATCG |
|  | Reverse: CCAGTTGGTAACAATGCCATGT |
| *DR5* (Mouse) | Forward: CTTTGCGTTGCTGCTTGCTGTG |
|  | Reverse: GGGTCCTCTTGATGGGCTCTCC |
| *Atoh1* (Mouse) | Forward: GAGTGGGCTGAAGTGAAGGAGTTG |
|  | Reverse: CGGGTAGACGGGATGCTCTCTC |
| *Gfi1* (Mouse) | Forward: AAGACCCTTTGCGTGCGAGATG |
|  | Reverse: GACAGCGTGGATGACCTCTTGAAG |
| *Spdef* (Mouse) | Forward: GGACGGATGGTGAGGTGGACTC |
|  | Reverse: TTGTTGAGCCAGCGGATGAAGC |
| *Tff3* (Mouse) | Forward: TGT CAG AGT GGA CTG TGG CTA CC |
|  | Reverse: GTT TGA AGC ACC AGG GCA CAT TTG |
| *Muc2* (Mouse) | Forward: TGCTGACGAGTGGTTGGTGAATG |
|  | Reverse: TGATGAGGTGGCAGACAGGAGAC |
| *TRAIL* (Mouse) | Forward: GACGCTTCCAAGATGGTCTCA |
|  | Reverse: AGCTCGAACAATCCTCCCTG |
| *Best2* (Mouse) | Forward: ACCAGTACGCCAGCCTCATCC |
|  | Reverse: CACGGTGCCAGCCACTATGC |
| *TBP* (Mouse) | Forward: ATCTTTAGTCCAATGATGCCTTACGG |
|  | Reverse: TTGCTGTTGCTGCTGCTGTC |
| *Tnf-α* (Mouse) | Forward: CGGGCAGGTCTACTTTGGAG |
|  | Reverse: ACCCTGAGCCATAATCCCCT |
| *Il-6* (Mouse) | Forward: TCCTTCCTACCCCAATTTCCA |
|  | Reverse: GTCTTGGTCCTTAGCCACTCC |
| *Il-1β* (Mouse) | Forward: TGCCACCTTTTGACAGTGATG |
|  | Reverse: TGATGTGCTGCTGCGAGATT |
| *GAPDH* (Human) | Forward: CACCCACTCCTCCACCTTTGAC |
|  | Reverse: GTCCACCACCCTGTTGCTGTAG |
| *DR5* (Human) | Forward: ACACAGCCACAATCAAGACTACGG |
|  | Reverse: GACTCCTGCCTCTCCCTGTTCTC |
| *TRAIL* (Human) | Forward: CGTGATCTTCACAGTGCTCCT |
|  | Reverse: CAGCTCGTTGGTAAAGTACACG |
| *BEST2* (Human) | Forward: TCCCGAACCGCTTACCCTCATC |
|  | Reverse: AGATTCTCCTCCTCCTCGCCAATAG |
| *TBP* (Human) | Forward: ACTCCACTGTATCCCTCCCC |
|  | Reverse: TATATTCGGCGTTTCGGGCA |
